# Radiation induces IRAK1 expression to promote radioresistance by suppressing autophagic cell death via decreasing the ubiquitination of PRDX1 in glioma cells

**DOI:** 10.1038/s41419-023-05732-0

**Published:** 2023-04-08

**Authors:** Jing Li, Yuchen Sun, Xu Zhao, Yuan Ma, Yuchen Xie, Siqi Liu, Beina Hui, Xiaobo Shi, Xuanzi Sun, Xiaozhi Zhang

**Affiliations:** 1 Department of Radiation Oncology, The First Affiliated Hospital of Xi’an Jiaotong University, Xi’an, 710061 China; 2 Department of Radiation Oncology, The Second Affiliated Hospital of Xi’an Jiaotong University, Xi’an, 710004 China

**Keywords:** Oncogenes, Ubiquitylation, Double-strand DNA breaks

## Abstract

Radiotherapy is the standard adjuvant treatment for glioma patients; however, the efficacy is limited by radioresistance. The function of Interleukin-1 receptor associated kinase 1 (IRAK1) in tumorigenesis and radioresistance remains to be elucidated. IRAK1 expression and its correlation with prognosis were analyzed in glioma tissues. We found that glioma patients with overexpressed IRAK1 show a poor prognosis. Notably, ionizing radiation (IR) remarkably induces IRAK1 expression, which was decreased by STING antagonist H-151 treatment. JASPAR prediction, ChIP assays, and dual luciferase reporter assays indicated that transcription factor FOXA2, suppressed by STING inhibition, directly binds to the IRAK1 promoter region and activates its transcription. IRAK1 knockdown inhibits malignancy and enhances the radiosensitivity of glioma in vitro and in vivo. To explore the potential IRAK1 interacting targets mediating the radioresistance of glioma cells, IP/Co-IP, LC-MS/MS, GST pull-down, and ubiquitination analyses were conducted. Mechanistically, IRAK1 bound to PRDX1, a major member of antioxidant enzymes, and further prevents ubiquitination and degradation of PRDX1 mediated by E3 ubiquitin ligase HECTD3; Both the DOC and HECT domains of HECTD3 directly interacted with PRDX1 protein. Overexpression of PRDX1 reverses the radiotherapy sensitization effect of IRAK1 depletion by diminishing autophagic cell death. These results suggest the IRAK1-PRDX1 axis provides a potential therapeutic target for glioma patients.

## Introduction

Glioma is the most prevalent intracranial primary malignancy, representing 75% of primary brain tumors in adults [1]. The World Health Organization (WHO) distinguishes gliomas into four grades, in which grade II and III tumors are described as lower-grade glioma (LGG), and grade IV tumors are commonly described as glioblastoma multiforme (GBM) [2]. Despite advances in treatment strategies, including surgery, radiotherapy, and chemotherapy, the outcomes of glioma patients remain unsatisfying, with a median survival time of no longer than 15 months in GBM patients [3]. Radiation is the most critical adjuvant treatment modality for newly diagnosed glioma patients following maximal safe surgical resection [4]. Still, due to the secondary radioresistance, the efficacy of ionizing radiation (IR) for glioma patients is modest, at best. Therefore, elucidating the underlying molecular mechanism involved in radioresistance may provide crucial insights into potential targets for radiosensitizing glioma.

As the first member discovered in the Interleukin-1 receptor associated kinase (IRAK) family, IRAK1 mediates Toll-like receptors (TLRs) and IL-1 receptor (IL-1R) signaling and plays a well-established role in innate immunity by inducing the production of type-I interferon (IFN) [5, 6]. IRAK1 is a serine/threonine-protein kinase that has been involved in tumorigenesis by driving TRAF6-mediated NF-kB and p38MAPK signaling activation in numerous cancers [6–9]. Also, IRAK1 augments cancer stemness and resistance to sorafenib treatment, the effective first-line multi-kinase inhibitor in hepatocellular carcinoma (HCC) [10]. Liu et al. reported that an IRAK1-PIN1 axis enhances intrinsic tumor resistance to radiation therapy in p53 mutant zebrafish, human cancer cell lines, and mouse xenografts [11]. However, very little is known about the effects of IRAK1 on tumor radioresistance and malignant behaviors in glioma.

Autophagy is a dynamic catabolic process that delivers damaged or excess proteins and organelles to lysosomes for degradation to maintain cellular homeostasis [12]. Autophagy induced by radiation is considered an efficient form of cell death [13]. Autophagic cell death is a form of regulated cell death that mechanistically depends on the autophagic machinery, displaying a large-scale accumulation of autophagosomes [14, 15]. Previous studies have demonstrated a synergistic effect between various drugs and radiation to promote autophagic cell death rather than apoptosis, contributing to the antitumor effect of radiotherapy in glioma [16]. Hence, autophagy may be a favorable option for potentiating the radiotherapy efficacy and as a radiosensitizer, which is undergoing preclinical trials [16, 17].

In the present study, we revealed that radiation remarkably induces IRAK1 expression and IRAK1 inhibition sensitizes glioma cells to radiation both in vitro and in vivo. We focused on the mechanism of the upregulated IRAK1 transcription after IR treatment in this research. In addition, we performed a comprehensive verification of the molecular mechanism of IRAK1 in regulating autophagy and its role in radiosensitivity.

## Materials and methods

### Cell culture

Glioma cell lines (U251, A172, U87, and T98G), 293T (HEK293T) cell line, and normal human astrocytes (NHA) were purchased from the China Center for Type Culture Collection (Shanghai, China). All the cells were cultured in DMEM medium (Gibco, CA, USA) containing 10% fetal bovine serum (FBS; Gibco, CA, USA) and 1% penicillin and streptomycin at 37 °C with 5% CO_2_ in a humidified atmosphere.

### Plasmid construction and reagents

The small interfering RNA (siRNA) for PRDX1, HECTD3, ATG5, FOXA2, and the negative control were designed and synthesized by GenePharma (Shanghai, China). The recombinant plasmids containing human Myc-PRDX1, Flag-IRAK1 (CMV-MCS-3FLAG-SV40-Puromycin), full-length Flag-HECTD3, Flag-DOC domain (amino acids 219-317), and Flag-HECT domain (amino acids 512-857) were acquired from GeneChem (Shanghai, China). The STING antagonist H-151 (T5674) and doxorubicin hydrochloride (T1020) were obtained from TargetMol (MA, USA). The proteasome inhibitor MG132 (S2619), protein synthesis inhibitor cycloheximide (CHX; S7418), and autophagy inhibitor 3-Methyladenine (3-MA; S2767) were purchased from Selleck Chemicals (Cambridge, UK).

### Cell transfection

The transfection of siRNA or plasmids was conducted using Lipofectamine 3000 reagent (Invitrogen, CA, USA) at a working concentration according to the manufacturer’s protocol. The lentiviruses delivering sh-IRAK1, and the corresponding control sequence were purchased from GeneChem (Shanghai, China). The target sequences of siRNA and shRNA are presented in Supplementary Table S2.

### Bioinformatic analysis

The glioma patients’ transcription profile and clinical data of the Cancer Genome Atlas (TCGA) cohort were downloaded from UCSC Xena (https://xena.ucsc.edu/). We obtained RNA-sequencing (RNA-seq) and survival data from 325 glioma cases in the Chinese Glioma Genome Atlas (CGGA) cohort and 475 glioma samples in the Rembrandt cohort from the CGGA (http://www.cgga.org.cn/index.jsp). A median cut-off value of IRAK1 expression was adopted to analyze the overall survival (OS) of the high- versus low-expression groups. The online tool GEPIA2 (http://gepia2.cancer-pku.cn/#index) was utilized to explore the correlation between IRAK1 and cGAS, STING mRNA expression levels in glioma patients from the TCGA dataset.

### Radiosensitivity signature

The radiosensitivity index (RSI) was developed to estimate the inherent radiosensitivity of a given tumor using a rank-based regression model. The RSI value was determined using the previously validated linear regression algorithm: RSI = −0.0098009 * AR + 0.0128283 * cJun + 0.0254552 * STAT1 - 0.0017589 * PKC - 0.0038171 * RelA + 0.1070213 * cABL - 0.0002509 * SUMO1 - 0.0092431 * PAK2 - 0.0204469 * HDAC1 - 0.0441683 * IRF1 [18]. A low RSI indicates high radiosensitivity, whereas a high RSI indicates high radioresistance in tumors.

### Clinical tissue specimens and Immunohistochemistry (IHC) staining

Glioma tissues (52 cases) and benign brain tumors (10 cases) were obtained from the First Affiliated Hospital of Xi’an Jiaotong University. All patients have signed the informed consent, and our study was approved by the Ethics Committee of the First Affiliated Hospital of Xi’an Jiaotong University. The IHC staining procedure was performed according to the standard avidin-biotin method described previously [19]. The IRAK1-stained sections were divided into high or low groups based on the intensity and extent of the staining. The antibodies used in IHC analysis are listed in Supplementary Table S4.

### Western blotting

Total protein was extracted with RIPA lysis buffer (Heart, Xi’an, China), and the lysates were then collected and centrifuged at 4 °C (12,000 rpm, 20 min). The proteins were separated on 10 or 12% SDS-PAGE gels (Beyotime, Shanghai, China) and transferred to PVDF membranes (Millipore, Billerica, MA, USA), which were incubated with primary antibodies at 4 °C overnight. After washing four times with TBST buffer, the membranes were incubated with HRP-conjugated secondary antibodies for 1 h at room temperature. The protein bands were visualized using chemiluminescence kits (Millipore, Billerica, MA, USA). The antibodies used to detect protein expression levels are listed in Supplementary Table S4.

### Quantitative real-time PCR (qRT-PCR)

Total RNA was isolated from cells using the Fastagen200 kit (Fastagen, Shanghai, China). Reverse transcription of RNA (1.0 μg) to cDNA was conducted by the PrimeScript RT Reagent Kit (TaKaRa, Osaka, Japan). Real-time PCR was carried out using SYBR Premix Ex Taq^TM^ II (TaKaRa, Osaka, Japan) with primers listed in Supplementary Table S3. Relative mRNA expression levels were quantified by applying the 2^−ΔΔCT^ method and normalized to those of GAPDH.

### Cell counting kit-8 (CCK-8) assay

Three thousand glioma cells per well in the exponential growth phase were seeded into 96-well plates (100 μL/well). At the indicated time of 6, 24, 48, 72, and 96 h after seeding, 10 μL CCK-8 solution (TargetMol, Boston, MA, USA) was added to each well and incubated for another 2 h. The optical density (OD, 490 nm) values were measured by a microplate reader.

### Transwell migration and invasion assays

Migration and invasion assays were performed using transwell chambers in 24-well plates (3422; Corning, NY, USA). For the migration assays, 600 μL of medium with 20% FBS was filled in the lower chambers. 200 μL of the serum-free medium containing 4 × 10^4^ glioma cells was added to the upper chambers and incubated at 37 °C for 16 h. For the invasion assays, Matrigel was spread on the upper chambers the day before cell seeding. About 600 μL of medium with 20% FBS was added to the bottom chamber, and 1.0 × 10^5^ cells in 200 μL of serum-free medium were seeded to the upper chamber and cultured for 24 h. At the end of incubation, the migrated and invaded glioma cells were fixed with 4% paraformaldehyde and stained with crystal violet. Images of stained cells were taken under a microscope, and the quantification was determined using ImageJ software.

### Clonogenic survival assay

Single-cell suspensions were inoculated into six-well plates at densities of 400–12,000 cells per well. After 12 h, cells were irradiated with six MV X-rays at gradient doses of 0, 2, 4, 6, and 8 Gy using an X-ray linear accelerator (Clinac 2100EX, Varian Medical Systems) with a 300 cGy/min dose rate and 100 cm source-surfaced distance. After 10–14 days of incubation, during which replaced the cell media every three days, the cells were rinsed with PBS, fixed with methanol, and stained with crystal violet. Colonies containing more than 50 cells were counted and analyzed based on the corresponding numbers of initial inoculating cells. The survival curves were fitted by GraphPad Prism 8 according to the Single-hit multitarget model: SF = 1 − (1 − e ^(−kD)^)^*N*^.

### Immunoprecipitation (IP)/co-immunoprecipitation assay (Co-IP)

Cell lysis buffer for Western and IP (Beyotime Biotechnology, Shanghai, China) supplemented with PMSF, protease inhibitor, and phosphatase inhibitor was employed to obtain cell lysates, which were then centrifuged at 12,000 rpm for 20 min. The antibodies adopted in IP /Co-IP experiments are listed in Supplementary Table S4. Pierce Crosslink Magnetic IP /Co-IP kits (Thermo Fisher Scientific, Waltham, MA) or Protein A/G PLUS-Agarose (Santa Cruz Biotechnology, Texas, USA) were applied for dragging out the antigen-antibody complex. At last, protein bound to the magnetic or agarose beads was extracted and resuspended in 2× loading buffer. The procedure of Western blotting assay was described above.

### Liquid chromatography tandem mass spectrometry (LC-MS/MS)

To investigate IRAK1-binding proteins in glioma cells, U251 cells were pretreated with MG132 (10 μM) for 4 h and then harvested using lysis buffer. The immunoprecipitates isolated by anti-IRAK1 and IgG control antibodies were analyzed using Dionex Ultimate 3000 RSLCnano (Buffer A: 0.1% formic acid solution; Buffer B: 0.1% formic acid + 80% acetonitrile solution; Thermo Scientific, USA). The peptides were then passed through a chromatographic column (Acclaim PepMap 75um X 150 mm,160321; Thermo) at a flow rate of 300 nL/min of Buffer A. After chromatographic separation, mass spectrometry analysis was performed based on a Q Exactive mass spectrometer (Thermo Scientific, USA) and MASCOT (http://www.matrixscience.com/).

### GST pull-down

*E. Coli* derived GST-Vector or GST-PRDX1 fusion protein was purified and prepared by Proteintech (Wuhan, China). Briefly, the total protein of 293T cells with or without Flag-tagged IRAK1 overexpression was incubated with GST-PRDX1 or GST-Vector fusion protein at 4 °C overnight. Then, cell lysates were incubated with glutathione-sepharose beads for 2 h. Beads were sufficiently washed with lysis buffer and boiled in SDS loading buffer for detecting the bound protein in vitro using western blotting assay.

### Chromatin immunoprecipitation (ChIP)—qPCR

ChIP was performed using the ChIP assay kit (Beyotime Biotechnology, Shanghai, China) according to the manufacturer’s instruction. Briefly, U251 (1 × 10^6^ cells) were cross-linked in 1% formaldehyde for 10 min at 37 °C, and then glycine solution was added to stop the reaction. After washing with pre-cold PBS buffer (supplemented with 1 mM PMSF), cells were centrifuged and lysed with SDS lysis buffer containing 1 mM PMSF. The chromatin was ultra-sonicated to fragments (~200–500 bp) 10 times with 10 s ultra-sonication at 10 s intervals. The lysates were subsequently incubated with IgG or ChIP-grade antibody against FOXA2 at 4 °C overnight and then incubated with Protein A + G Agarose/Salmon Sperm DNA at 4 °C for 3 h. After washing with low salt and high salt buffer, elution, and reverse cross-linking, the DNA was added with EDTA, Tris pH 6.5, and proteinase K at 45 °C for 1 h and then was purified for qPCR analysis. The primer sequences used for ChIP-qPCR assay in the IRAK1 promotor region were provided in Supplementary Table S3, and the antibodies were listed in Supplementary Table S4.

### Luciferase reporter assay

To investigate the effect of FOXA2 on the activation of IRAK1 promotor, the construct or truncated promotor region of the IRAK1 genes was cloned into the upstream of the luciferase reporter gene of the pGL3.0 Basic vector (GenePharma, Shanghai, China). U251 cells (1 × 10^5^) were seeded in a 24-well plate and cultured for 24 h. Then, cells were co-transfected with the wide-type or mutant luciferase plasmids, pRL-TK plasmid, and FOXA2 siRNA/ negative control using Lipo3000^TM^ transfection reagent. After transfection for 48 h, the luciferase activity was measured with the Dual Luciferase Reporter Gene Assay Kit (Beyotime Biotechnology, Shanghai, China). The activity of Firefly luciferase activity was normalized to the Renilla luciferase activity as control.

### Immunofluorescence staining

U251 and A172 cells were seeded on 20 × 20 mm glass-bottom round dishes (Corning, NY, USA) and cultured overnight to 50% confluence. Cells were fixed with 4% paraformaldehyde for 20 min and subsequently permeabilized with 0.1% Triton X-100 before being blocked by 5% bovine serum albumin for 30 min at room temperature. The primary antibodies were added to glioma cells and incubated at 4 °C overnight. After washing with PBS, cells were incubated with secondary antibodies for 1 h away from light. Antifade Mounting Medium with DAPI (Beyotime Biotechnology, Shanghai, China) was utilized to stain nuclei for 10 min, and then a Leica TC5 SP5 confocal microscope was adopted to visualize and image the cells. Antibodies are listed in Supplementary Table S4.

### Flow cytometry

The cell cycle kit (KeyGEN, Nanjing, China) and Annexin V-APC/7-AAD apoptosis kit (BD Pharmingen, New Jersey, USA) were utilized to detect the cell cycle distribution and apoptosis rates, respectively. The procedure was performed according to a previous description [20].

### Alkaline comet assay

DNA damage was detected with a comet assay kit (KeyGEN, Nanjing, China) under alkaline conditions. In brief, cells were harvested and washed with cold PBS buffer at indicated time points after 8 Gy X-ray irradiation. Cell concentration was adjusted to 1 × 10^6^ cells /mL using PBS. Then, mixed 10 μL glioma cells with 75 μL 0.7% low melting point agarose and plated the mixture on pre-coated normal melting point agarose slides. Slides were placed in pre-cooled lysis buffer at 4 °C for 2 h and transferred to the alkaline electrophoresis solution (1 mM EDTA, 300 mM NaOH) in a horizontal electrophoresis tank for 40 min at room temperature. Subsequently, the slides were electrophoresed at 25 V for 30 min and neutralized three times with 0.4 mM Tris-HCl (pH7.5) at 4 °C for 10 min each. Cells were finally stained with 20 μL Propidium Iodide for 10 min in the dark and observed under a Leica inverted fluorescence microscope. DNA damage was quantified by the comet tail intensity (tail DNA%) using Comet Assay Software Project (CASP, version 1.2.2).

### Xenograft tumor mouse model and treatment

The BALB/C nude mice (4 weeks, female) were purchased from the Experimental Animal Center of Xi’an Jiaotong University and housed in a specific-pathogen-free animal room. Mice were divided into four groups (5 mice/group) using a computer-based random order generator. U251 cells suspended in Matrigel were subcutaneously injected into the right blanks of mice (5 × 10^6^ cells/ 100 μL). The tumor length (a) and width (b) were measured using calipers every 3 days from the seventh day after tumor cells injection. Tumor volume was calculated with the following formula: *V* = *ab*^2^/2. After the diameter of the tumors reached 5 mm, the mice were exposed to 10 Gy radiation targeted to the tumor region, while the control groups were not irradiated. On day 28 after injection, the mice were humanely sacrificed, and tumor samples were collected and paraffin-embedded for the subsequent IHC analyses. The animal experiment in this study was approved by the Institution Animal Care and Use Committee of the First Affiliated Hospital of Xi’an Jiaotong University.

### Statistical analysis

All the experiments were performed independently in triplicate at least. Data were analyzed by GraphPad Prism 8 software and presented as the mean ± SD. Statistical *p* values were determined by two-tailed Student’s t-test or one-way ANOVA analyses. Survival curves were constructed by the Kaplan-Meier method and the log-rank test. The association of the expression was evaluated by the Spearman correlation. *p* < 0.05 was considered statistically significant.

## Results

### IRAK1 is upregulated in glioma and associated with poor prognosis

**Fig. 1:**
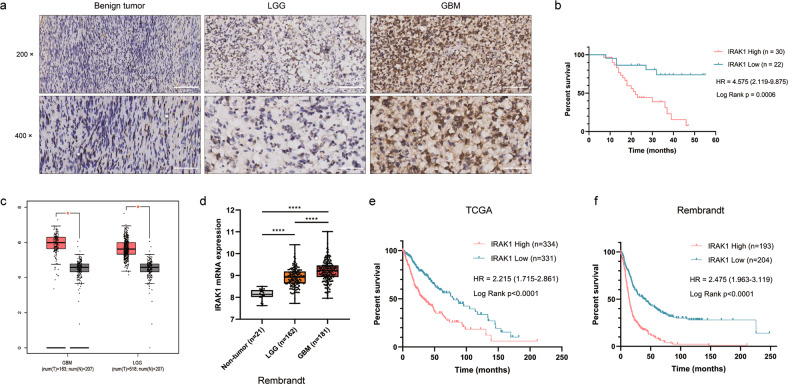
The upregulated expression of IRAK1 is correlated with poor prognosis in glioma patients. **a** Representative IHC images of IRAK1 protein in human glioma tissues and brain benign tumor tissues. 200×: scale bar = 100 μm; 400×: scale bar = 50 μm. **b** Kaplan-Meier survival plot for overall survival grouped by IRAK1 expression in 52 glioma patients (*p* = 0.0006). **c** IRAK1 mRNA expression profiling data from TCGA and GTEx databases analyzed by the GEPIA webserver (Non-tumor, n = 207; LGG, *n* = 518; GBM, *n* = 163; *p < 0.05). **d** IRAK1 expression analysis for non-tumor and glioma samples in Rembrandt database (****p < 0.0001). The correlation between IRAK1 and OS in glioma patients from TCGA (**e**) and Rembrandt (**f**) cohorts, with the hazard ratio (HR) and *p* values displayed.

To investigate the role of IRAK1 played in glioma, we first assessed its expression levels in glioma and benign brain tumor tissues by IHC analysis. As shown in Fig. 1a and Table 1, IRAK1 expression was much upregulated in glioma tissues in comparison with benign brain tumor tissues. Moreover, the Spearman correlation analysis between IRAK1 expression pattern and clinical characteristics of glioma patients was conducted. As illustrated in Table 2, the result verified that IRAK1 expression level has a significantly positive association with tumor recurrence (*p* = 0.001) and higher pathological grade (*p* = 0.037), suggesting that IRAK1 might play a tumor-promoting role in glioma. Meanwhile, glioma patients with higher expression of IRAK1 exhibited a shorter overall survival (Fig. 1b). Similar to the observation in the glioma cohort from our department, IRAK1 mRNA expression was higher in glioma, including LGG and GBM, as compared to nontumor tissues in TCGA (Fig. 1c) and Rembrandt (Fig. 1d) databases. The Kaplan-Meier survival analysis from TCGA and Rembrandt datasets, showing a worse prognosis of glioma patients with higher IRAK1 expression, further indicated the correlation of IRAK1 in glioma development (Fig. 1e, f).

**Table 1:** IRAK1 expression patterns in glioma and benign tumor tissues derived from IHC analysis.

IRAK1 expression	Glioma tissue		Benign tumor tissue	
	Cases	Percentage	Cases	Percentage
Low	22	42.30%	10	100%
High	30	57.70%	0	-

*P* = 0.003.

**Table 2:** Relationship between IRAK1 expression level and clinical characteristics in glioma patients.

Features	No. of patients	IRAK1 expression		*P* value
		low	high	
All patients	52	22	30	
*Age*				0.253
<42	19	10	9	
≥42	33	12	21	
*Gender*				0.148
Male	25	8	17	
Female	27	14	13	
*Tumor recurrence*				**0.001**
No	24	16	8	
Yes	28	6	22	
*Grade*				**0.037**
I	1	1	0	
II	17	11	6	
III	23	8	15	
IV	11	2	9	

### IRAK1 knockdown inhibits the tumorigenesis of glioma in vitro

**Fig. 2:**
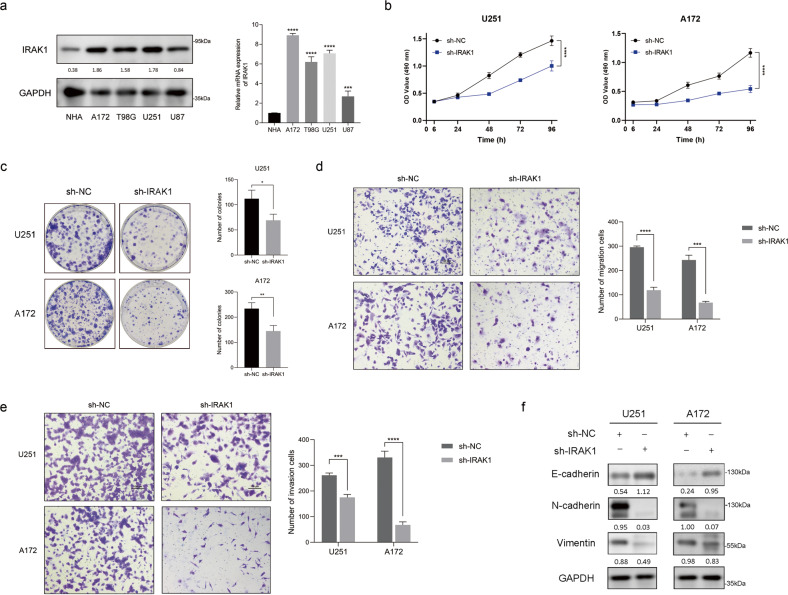
IRAK1 knockdown inhibits glioma malignancy in vitro. **a** Western blotting analysis and qRT-PCR assay of IRAK1 expression in NHA cells and human glioma cell lines, including A172, T98G, U251, and U87 cells. **b** CCK-8 proliferation assay transfected with sh-NC or sh-IRAK1 in U251 and A172 cells. **c** Colony formation assay to detect the effect of IRAK1 knockdown on the proliferation of glioma cells. Representative images and the comparison on cell migration (**d**) and invasion (**e**) ability demonstrated by Transwell assay between IRAK1 knockdown cells and the control ones based on U251 and A172 cells. Scale bar = 100 μm. **f** The protein expression of EMT-related markers, including E-cadherin, N-cadherin, and Vimentin, was determined by Western blotting in sh-IRAK1 and sh-NC glioma cells. The ratio of the target proteins to GAPDH in the form of grayscale value was shown below each lane. All data were presented as mean ± SD from three independent experiments. *p < 0.05, **p < 0.01, ***p < 0.001, ****p < 0.0001.

To gain a deeper insight into the biological function of IRAK1 in glioma, we detected the endogenous expression of IRAK1 in four human established glioma cell lines and NHA cells. Western blotting and qRT-PCR analyses showed that the protein and transcript levels of IRAK1 were obviously higher in all four glioma cell lines compared to NHA cells (Fig. 2a). Cell lines U251 and A172 were selected for the subsequent exploration as they possessed the highest expression of IRAK1. We, therefore, built IRAK1-knockdown glioma cell models based on U251 and A172 cells. Following the transfection of sh-IRAK1 or sh- negative control (NC) lentivirus, the efficiency was verified with Western blotting and qRT-PCR assays, suggesting the successful construction of stable IRAK1-knockdown glioma cell models (Supplementary Fig. S1a).

To explore the effect of IRAK1 in glioma malignancy in vitro, CCK-8 proliferation assay, colony formation assay, and transwell migration and invasion assays were performed. As displayed in Fig. 2b, c, knockdown of IRAK1 significantly repressed cell viability and colony formation capacity of U251 and A172 cells. Meanwhile, transwell assays revealed that IRAK1 silencing could remarkably impede the migratory and invasive ability of glioma cells (Fig. 2d, e). Epithelial mesenchymal transition (EMT), a process by which epithelial cells acquire mesenchymal phenotypes, confers metastatic properties [21]. Tumor cells undergoing EMT exhibit molecular alterations, as demonstrated by the loss of epithelial marker E-cadherin, and the gain of mesenchymal markers, including N-cadherin and Vimentin [22]. Our results showed that the facilitation of IRAK1 on glioma cell migration and invasion could be partially explained by the upregulated expression of E-cadherin and the downregulated expression of N-cadherin and Vimentin (Fig. 2f).

### IR induces IRAK1 expression by activating STING-FOXA2-initiated IRAK1 transcription

**Fig. 3:**
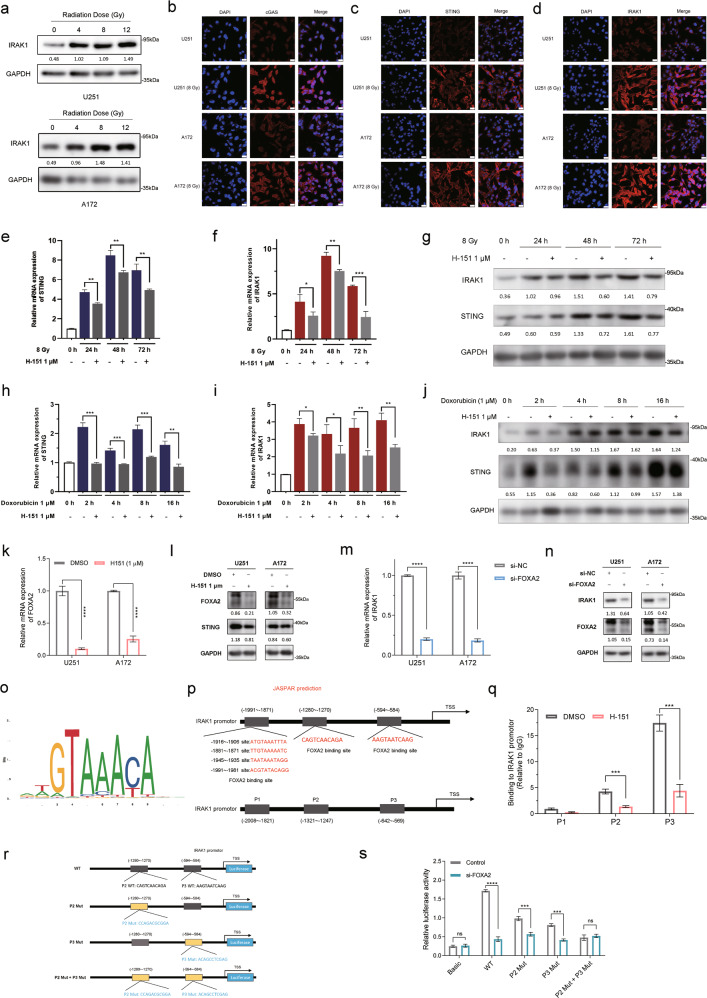
cGAS-STING signaling participates in radiation-induced IRAK1 expression. **a** U251 and A172 glioma cells were exposed to a range of X-ray IR doses. IRAK1 protein expression levels were detected by Western blotting at 48 h post IR exposure. Immunofluorescence staining was performed to determine the expression abundance of cGAS (**b**), STING (**c**), and IRAK1 (**d**) in response to 8 Gy IR. Scale bar = 25 μm. qRT-PCR measurement of STING (**e**) and IRAK1 (**f**) mRNA expression in U251 cells treated with 8 Gy X-ray irradiation ± H-151 (1 μM). **g** The level of IRAK1 and STING assessed by Western blotting analysis in U251 cells at 0, 24, 48, and 72 h after 8 Gy irradiation ± H-151 (1 μM). qRT-PCR measurement of STING (**h**) and IRAK1 (**i**) mRNA expression in U251 cells treated with doxorubicin (1 μM) ± H-151 (1 μM). **j** Effects of doxorubicin (1 μM) ± H-151 (1 μM) treatment on IRAK1 and STING protein expression were evaluated by Western blotting in U251 cells. The influence of H-151 (1 μM) on FOXA2 was measured by RT-qPCR (**k**) and Western blotting (**l**). The changes of the mRNA (**m**) and protein levels (**n**) of IRAK1 caused by FOXA2 knockdown in glioma cells. The gray value ratios of the corresponding proteins /GAPDH were shown under the lanes. **o, p** The FOXA2-binding motif was predicted by JASPAR, and schematic images of the potential FOXA2 binding sites in the IRAK1 promoter region are shown. **q** ChIP-qPCR analysis of FOXA2 binding on IRAK1 promoter in U251 cells. **r**, **s** Luciferase reporter assay for detecting the activity of wild-type or mutant IRAK1 promoters in U251 cells which were transfected with FOXA2 siRNA or Scramble siRNA. Data were shown as mean ± SD from three independent experiments. *p < 0.05, **p < 0.01, ***p < 0.001.

As radiation is a principal adjuvant therapy for treating glioma, we next measured the protein expression of IRAK1 in cells that received different doses of X-ray irradiation (IR). Western blotting analysis revealed that IR exposure increased IRAK1 expression levels dose-dependently in U251 cells. Likewise, IRAK1 expression was markedly upregulated following IR compared with non-irradiated cells (0 Gy) serving as a baseline in A172 cells, reaching a peak at 8 Gy treatment (Fig. 3a). It was reported that cyclic GMP-AMP (cGAMP) synthase (cGAS), a cytosolic nucleic acid sensor, can be activated by double-stranded DNA (dsDNA) accumulated in irradiated cytoplasm [23, 24]. cGAS activation generates the second messenger cGAMP, bounds to and activates Stimulator of Interferon Genes (STING) [25]. IR causes DNA damage through direct and indirect effects and induces dsDNA accumulation in the cytoplasm, which can be sensed by cGAS/STING signaling. Activated STING triggers the phosphorylation of interferon regulator factor 3 (IRF3), which then translocases to the nucleus as a transcription factor to induce the production of type-I IFN and NF-κB for activation [26]. Additionally, as a widely accepted upstream component of the NF-κB signaling cascades, IRAK1 interacts with TNF receptor-associated factor 6 (TRAF6) to promote IFN-β production and the activation and nuclear translocation of NF-κB [27]. Therefore, STING and IRAK1 are both indispensable upstream molecules in the induction of type-I IFN and activation of NF-κB. Based on the above similar roles of IRAK1 and STING, and STING can be dynamically activated in response to IR, we hypothesized whether STING signaling participates in IR-induced IRAK1 upregulation in glioma cells. Indeed, the activation of cGAS and STING stimulated by IR treatment was confirmed by immunofluorescence staining (Fig. 3b, c). Consistently, IRAK1, located mainly in the cytoplasm, was upregulated upon irradiation in U251 and A172 cells (Fig. 3d). We next explored the correlation for IRAK1 expression with cGAS and STING based on glioma patients from TCGA datasets. As shown in Supplementary Fig. S1b, IRAK1 expression had a significantly positive correlation with the mRNA expression levels of cGAS (*R* = 0.51, *p* = 2.2e-46) and STING (*R* = 0.56, *p* = 9.5e-57), suggesting cGAS-STING signaling may mediated IR-induced IRAK1 expression.

Therefore, we utilized H-151, a STING inhibitor, for the subsequent investigation [28]. Cellular inhibitory activities of H-151 on STING post irradiation (8 Gy) at various time points were verified via RT-qPCR and Western blotting in U251 (Fig. 3e, g) and A172 cells (Supplementary Fig. S1c, e). Our results showed that STING inhibition significantly attenuated IR-induced IRAK1 expression at both transcriptional and translational levels (Fig. 3f, g, Supplementary Fig. S1d, e). Since the major cellular effect of irradiation is to trigger DNA damage responses, we probed whether this conclusion also applies to doxorubicin, a topoisomerase II poison and DNA intercalator that generates DNA damage [29]. Analogously, doxorubicin-induced expression of IRAK1 could be remarkably reduced by H-151 treatment in U251 (Fig. 3h–j) and A172 cells (Supplementary Fig. S1f-h). These results demonstrated that the activation of cGAS-STING signaling, at least partially, mediates the upregulated mRNA and protein expression levels of IRAK1 induced by irradiation or doxorubicin treatment.

Next, we sought to decipher the transcription factor that may be responsible for the upregulation of IRAK1 stimulated by IR-induced STING activation. Qiao et al. recently performed ATAC-seq analysis in STING-βKO mice and identified chromatin regions exhibiting accessibility changes by STING inactivation, and known motif enrichment on STING-dependent differential peaks showed that the binding site for transcription factor Forkhead box protein A2 (FOXA2) was significantly enriched with the lowest *P* value [30]. Therefore, RT-qPCR and Western blotting analysis were performed to validate that STING inhibitor H-151 significantly suppressed the mRNA and protein levels of FOXA2 in glioma cells (Fig. 3k, l). Furthermore, knockdown of FOXA2 resulted in IRAK1 downregulation, in both mRNA and protein levels (Fig. 3m, n). We identified whether FOXA2 directly regulated IRAK1 transcription. The prediction of FOXA2 binding sites in the IRAK1 promoter region using the JASPAR website (http://jaspar.genereg.net/) revealed three putative motifs (P1-P3), which had high scores, in the IRAK1 promoter (Fig. 3o, p). Further, ChIP-qPCR confirmed that FOXA2 is recruited to the P2 (−1321~−1247 bp) and P3 (−642~−569 bp) sites of the IRAK1 promoter region, rather than the P1 site. Meanwhile, the binding of FOXA2 to P2 and P3 sites was impaired following H-151 treatment, suggesting that STING inhibition markedly decreased the FOXA2-dependent transcriptional activities of IRAK1 (Fig. 3q). Then, luciferase reporter gene plasmids containing the wild type (WT), P2 mutant (P2 Mut), P3 mutant (P3 Mut), and combined mutant sites (P2 Mut + P3 Mut) were cloned into pGL3.0 vector (Fig. 3r), and the results of dual-luciferase reporter assay showed that silencing FOXA2 decreased the WT, alone P2 Mut or P3 Mut IRAK1 reporter activity, however, the luciferase activity of the P2 Mut + P3 Mut IRAK1 was not affected by FOXA2 knockdown (Fig. 3s). It suggested that both the sites P2 and P3 of IRAK1 promoter were the binding regions combined with FOXA2. Collectively, these data suggested that IR induces IRAK1 expression by activating STING-FOXA2-initiated IRAK1 transcription.

### IRAK1 inhibition enhances the radiosensitivity of glioma cells

**Fig. 4:**
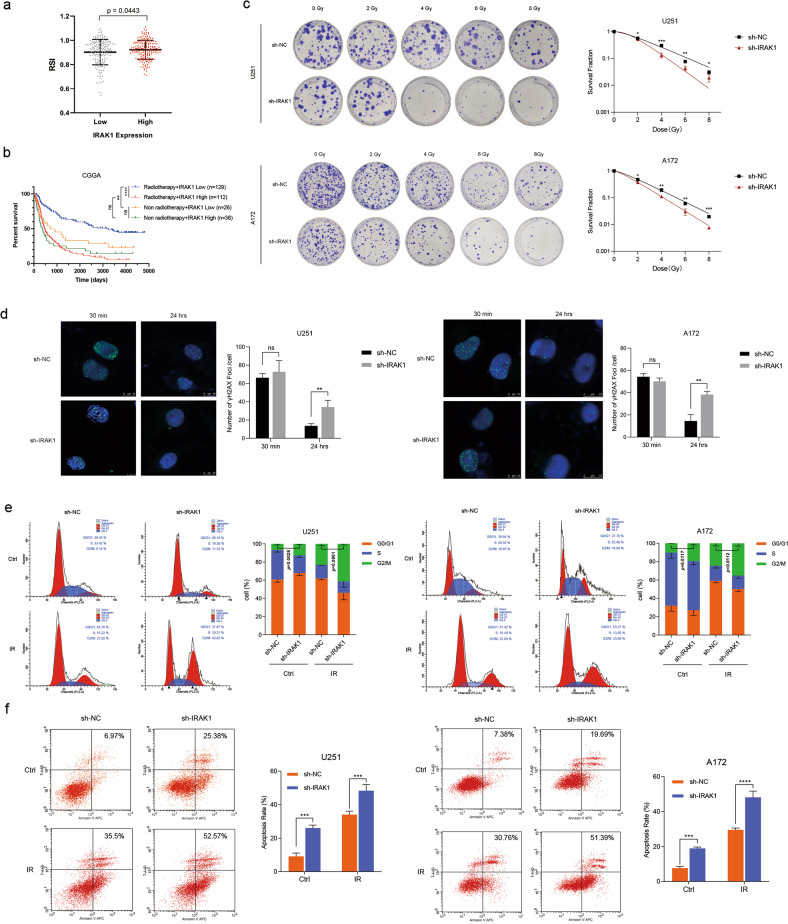
Knockdown of IRAK1 enhances the radiosensitivity of glioma cells. **a** For glioma patients from the TCGA dataset, comparison of RSI between IRAK1 low and IRAK1 high expression groups, divided by the median cut-off value (*p* = 0.0443, with two-tailed Student’s *t* test). RSI, radiosensitivity index. **b** Kaplan-Meier OS analysis of glioma patients based on IRAK1 expression and with/without radiotherapy in CGGA database. **c** Clonogenic assays and the corresponding survival fraction curves of U251 and A172 cells stably transfected with IRAK1 knockdown or negative control lentivirus following exposure to 0, 2, 4, 6, 8 Gy of X-rays. **d** Formation of γ-H2AX foci at 30 min and 24 h after exposure to 8 Gy IR, which was confirmed by immunofluorescence staining in U251 and A172 cells transfected with sh-IRAK1 or sh-NC. Scale bar, 10 μm. Cell cycle distribution (**e**) and apoptosis rates (**f**) of U251 and A172 cells transduced with sh-IRAK1 or sh-NC with or without exposure to 8 Gy IR. Flow cytometry was utilized to detect the cell cycle distribution at 12 h post IR and the apoptosis rates at 6 h post IR. Data were presented as mean ± SD; *n* = 3 independent experiments. *p < 0.05, **p < 0.01, ***p < 0.001, ****p < 0.0001.

RSI was previously validated to be a valuable score in guiding radiotherapy decisions in a number of malignancies. Tumors presenting higher RSI were more radioresistant [31]. To understand the role of IRAK1 in glioma radioresistance, we preliminarily calculated the RSI based on the expression profiling of glioma samples from the TCGA cohort. The results showed that glioma patients with high IRAK1 expression had higher RSI, indicating more resistance to radiation therapy (Fig. 4a). Furthermore, survival curves of the CGGA database were conducted grouped by IRAK1 expression and with/without radiotherapy. As depicted in Fig. 4b, for glioma patients who received radiotherapy, the IRAK1 low expression group exhibited an obvious survival advantage compared with IRAK1 high expression ones. Notably, no statistical difference was identified in the survival time between patients with IRAK1 high expression who underwent radiotherapy and non-radiotherapy. The analyses indicated that glioma patients with high IRAK1 expression could not benefit from radiotherapy.

We next performed clonogenic survival assays to further investigate the effect of IRAK1 after DNA damage in glioma cells. We found that IRAK1 inhibition markedly impeded the survival fraction of the U251 and A172 cells subjected to different doses of IR (Fig. 4c, Supplementary Table S1). IRAK1 knockdown and negative control cells were irradiated at a dose of 8 Gy and stained for the presence of γ-H2AX foci, an established marker of double-stranded DNA breaks (DSB) within the nucleus [32]. The γ-H2AX foci accumulated rapidly and reached a peak at 30 min post IR; the foci remaining at 24 h represented persistent DNA damage suggesting enhanced radiosensitivity [33]. IRAK1 inhibition cells demonstrated similar counts of γ-H2AX foci per nucleus compared to control cells at 30 min after IR. Remarkably, an increasing number of foci were detected at 24 h post IR in IRAK1 silencing glioma cells, indicating persistent DNA damage caused by IRAK1 knockdown (Fig. 4d). Cell cycle arrest is one of the most common effects of IR. The higher proportion of cells in the G2/M phase suggests being more sensitive to IR [34]. Cell cycle distribution analysis showed that the combination of IR and IRAK1 knockdown significantly induced the G2/M arrest of glioma cells (Fig. 4e). Additionally, IRAK1 inhibition increased the apoptosis rates of irradiated glioma cells detected by flow cytometry (Fig. 4f). These results elucidated that IRAK1 knockdown enhances the cell-killing effect of IR and sensitizes glioma cells to IR.

### IRAK1 effectively prevents PRDX1 degradation by reducing its ubiquitination

**Fig. 5:**
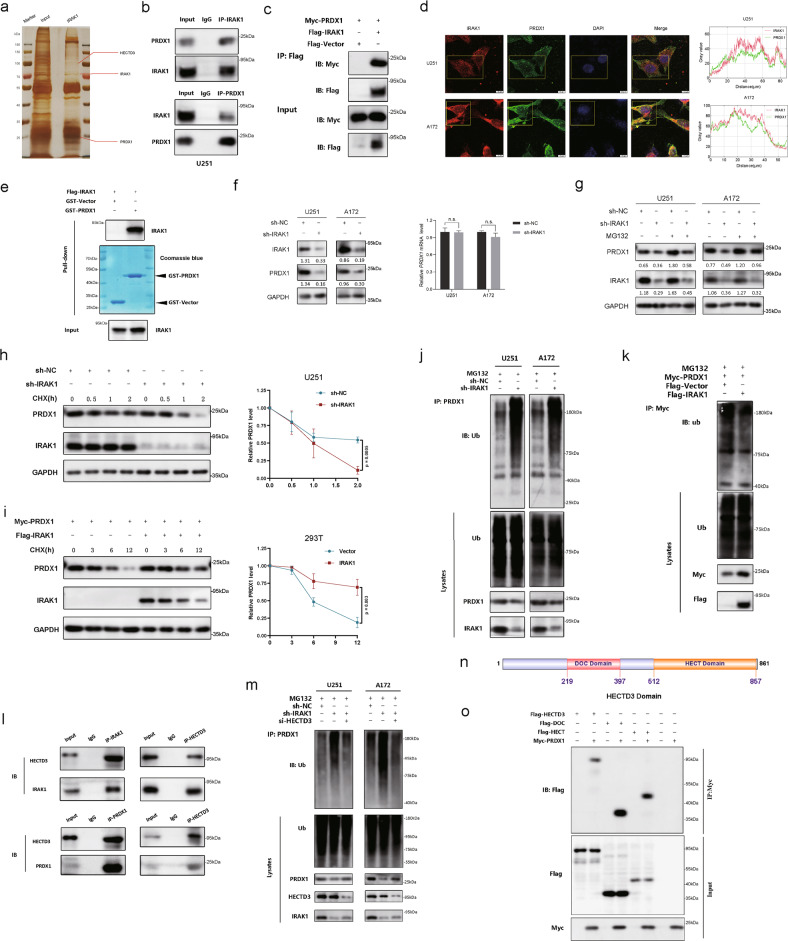
IRAK1 prevents the degradation of PRDX1 by decreasing its ubiquitination mediated by E3 ligase HECTD3. **a** Silver-stained SDS-PAGE gels showed the IRAK1-immunoprecipitated proteins separated from U251 cells. Red lines indicated the proteins of interest. **b** Co-IP detected the interaction of endogenous IRAK1 and PRDX1 in U251 cells. **c** Flag-IRAK1 and Myc-PRDX1 plasmids were transiently transfected into 293T cells, and Co-IP assays were performed to detect the exogenous interaction of IRAK1 and PRDX1. **d** Immunofluorescence staining revealed the cellular location of IRAK1 and PRDX1 in U251 and A172 cells, quantitatively analyzed by ImageJ software. Scale bar, 10 μm. **e** Flag-IRAK1 and GST-PRDX1 proteins were prepared in 293 T cells and GST pull-down assays were conducted. Bacterially expressed GST-PRDX1 protein was purified and confirmed by Coomassie blue staining. **f** The protein and mRNA expression of PRDX1 after IRAK1 knockdown were detected by Western blotting and qRT-PCR assays, respectively. **g** The effect of MG132 treatment in U251 and A172 cells transfected with sh-IRAK1 lentivirus. Relative gray intensity to GAPDH was presented below the lanes. The effect of CHX treatment (100 μg/mL) and greyscale quantification analysis in sh-NC or sh-IRAK1 U251 cells (**h**), as well as in 293T cells transfected with Flag-IRAK1 and Myc-PRDX1 or the empty vector plasmids (**i**). Analysis of PRDX1 ubiquitination upon IRAK1 knockdown in glioma cells (**j**), and IRAK1 overexpression in 293 T cells (**k**) by Western blotting with indicated antibodies. **l** Co-IP assay detecting the endogenous interaction of IRAK1 and PRDX1 with HECTD3 in U251 cells. **m** Co-IP assay detecting the effects of HECTD3 knockdown on the ubiquitination and stability of PRDX1 protein in sh-NC or sh-IRAK1 glioma cells treated with MG132. **n** DOC (219-397 aa) and HECT (512-857 aa) domains diagram of HECTD3 drawn by IBS 1.0 (http://ibs.biocuckoo.org/online.php). **o** HECTD3 full-length and truncations plasmids were co-transfected with Myc-PRDX1 plasmid into 293T cells and Co-IP assays were performed using anti-Myc antibody. 293T cells transfected with Myc-PRDX1 alone or Flag-tagged truncations of HECTD3 in absence of myc-PRDX1 were set as negative controls.

To determine the molecular mechanism by which IRAK1 promotes glioma radioresistance, IP combined with LC-MS/MS was then performed. By screening the potential interacting proteins with IRAK1, we noticed a candidate interaction protein Peroxiredoxin 1 (PRDX1, 23 kDa; Fig. 5a, Supplementary Fig. S2a). As a major member of antioxidant enzymes, PRDX1 was previously reported to effectively eliminate the accumulation of reactive oxygen species (ROS) [35]. Interestingly, knockdown of IRAK1 also significantly increased ROS levels in glioma cells (Supplementary Fig. S2b). Co-IP verified the endogenous and exogenous interaction between IRAK1 and PRDX1 (Fig. 5b, c, Supplementary Fig. S2c). Immunofluorescence staining further demonstrated the co-localization of IRAK1 and PRDX1 mainly in the cytoplasm (Fig. 5d). We then performed GST-pulldown assay using the purified GST-PRDX1 protein and the cellular extract lysates prepared from 293T cells transfected with Flag-IRAK1 plasmid. As shown in Fig. 5e, the results confirmed that GST-PRDX1 efficiently co-eluted with IRAK1, indicating a direct interaction between IRAK1 and PRDX1.

Since IRAK1 interacted with PRDX1, we tried to figure out whether IRAK1 is involved in regulating PRDX1 expression. The PRDX1 protein level was markedly downregulated after IRAK1 knockdown in Western blotting assay, whereas the mRNA expression level of PRDX1 was not affected (Fig. 5f). Also, the death domain and TRAF6-binding motif of IRAK1 was reported to induce TRAF6 degradation [36]. Therefore, we suspected that the regulation of PRDX1 by IRAK1 is likely at the posttranscriptional level. Subsequently, we detected the effects of IRAK1 on the degradation of PRDX1 protein by using the protein synthesis inhibitor CHX. The results showed that knockdown of IRAK1 significantly promoted the endogenous degradation of PRDX1 (Fig. 5h, Supplementary Fig. S2d), and overexpression of IRAK1 inhibited the degradation of the exogenous PRDX1 after treatment with CHX (Fig. 5i). These results indicated that IRAK1 knockdown could notably shorten the half-life of the PRDX1 protein. Ubiquitination is a common post-translational modification to add ubiquitin (8.5 kDa) to the substrate protein. The ubiquitin-proteasome system (UPS) is responsible for the specific degradation of more than 80% of intracellular proteins. Therefore, we explored whether the UPS mediates the degradation of PRDX1 regulated by IRAK1. We then treated glioma cells with the proteasome inhibitor MG132 and found that IRAK1 silencing-mediated destabilization of PRDX1 was reversed by MG132 (Fig. 5g). The results demonstrated that IRAK1 knockdown downregulates PRDX1 protein level through the ubiquitin-proteasome pathway. We, therefore, examined the effects of IRAK1 on the ubiquitination of PRDX1. As might be expected, knockdown of IRAK1 significantly increased the ubiquitination of PRDX1 (Fig. 5j), and overexpression of IRAK1 reduced the ubiquitination level of PRDX1 (Fig. 5k). These results suggested that IRAK1 prevents the degradation of PRDX1 protein via the ubiquitin-proteasome pathway.

### IRAK1 inhibits E3 ligase HECTD3-mediated PRDX1 degradation

The above results showed that IRAK1 prevents the ubiquitination and degradation of PRDX1. The key to the selective degradation mechanism of the UPS is E3 ubiquitin ligase, which recognizes the degraded protein and adds ubiquitin to the substrate. Thus, we hypothesized that specific E3 ubiquitin ligase might be involved in catalyzing the ubiquitination of PRDX1. We then found the E3 ligase HECTD3 by UbiBrowser (Supplementary Fig. S3a, http://ubibrowser.bio-it.cn/ubibrowser/) and LC-MS/MS (Supplementary Fig. S3b). Consistent with our hypothesis, endogenous Co-IP assay validated that both IRAK1 and PRDX1 protein interacted with HECTD3 (Fig. 5l). Further, MG132 and CHX treatment demonstrated that depletion of HECTD3 abolished the inhibitory effects of sh-IRAK1 on the protein level of PRDX1 (Supplementary Fig. S3c–f). Co-IP implied that HECTD3 silencing ameliorated the promoting effect of IRAK1 knockdown on the ubiquitination and degradation of PRDX1 protein (Fig. 5m). These results hinted that the E3 ligase HECTD3 mediates the ubiquitination of PRDX1 resulting from IRAK1 knockdown in glioma cells. As shown in Fig. 5n, HECTD3 contains DOC domain and HECT domain. Based on this, Flag-tagged DOC domain (219-397 aa), HECT domain (512-857 aa), and full-length Flag-tagged HECTD3 plasmids were constructed for transient transfection. Co-IP experiments showed that exogenous Flag-HECTD3, Flag-DOC, and Flag-HECT were pulled down by Myc-PRDX1, indicating that both full-length HECTD3 protein and isolated DOC and HECT domains of HECTD3 interact with PRDX1 (Fig. 5o).

### Overexpression of PRDX1 reverses the radiosensitivity resulting from IRAK1 depletion in vitro

**Fig. 6:**
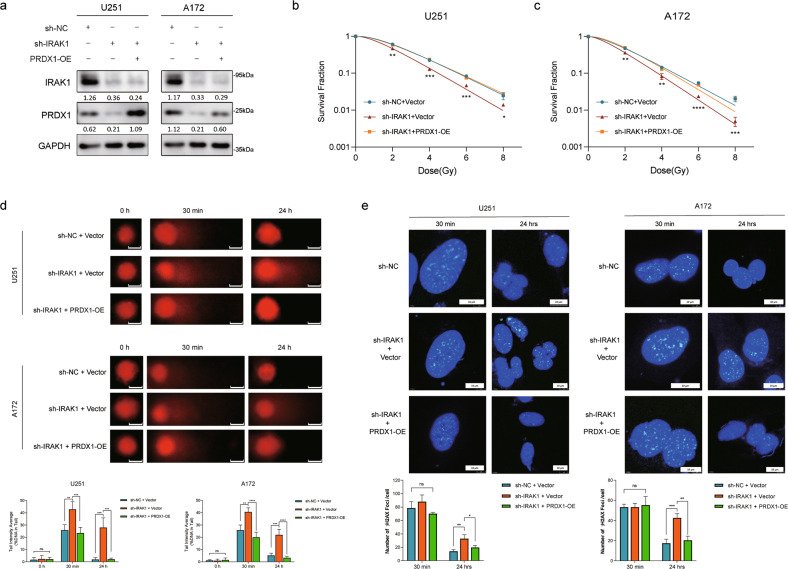
Overexpression of PRDX1 reverts the radiosensitivity caused by IRAK1 depletion in vitro. **a** U251 and A172 cells silencing IRAK1 were transfected with PRDX1 overexpression (PRDX1 OE) or Vector plasmids, and the expression of IRAK1 and PRDX1 was verified by Western blotting. Relative protein abundance of each blot was normalized to the gray value of GAPDH. Survival fraction curves of sh-NC + Vector, sh-IRAK1 + Vector, and sh-IRAK1 + PRDX1 OE groups in U251 (**b**) and A172 cells (**c**) following exposure to 0, 2, 4, 6, 8 Gy of X-rays. **d** Representative images of comet assay and quantitative analysis of tail intensity for 8 Gy IR-induced DNA damage in the indicated groups of U251 and A172 cells. Scale bar, 10 μm. **e** Representative images and quantitative analysis of the counts of γ-H2AX foci at 30 min and 24 h after exposure to 8 Gy IR, which was confirmed by immunofluorescence staining in the indicated grouped U251 and A172 cells. Scale bar, 10 μm. Data were presented as the mean ± SD; *n* = 3 independent experiments. *p < 0.05, **p < 0.01, ***p < 0.001, ****p < 0.0001.

To validate whether knockdown of IRAK1 downregulates PRDX1 and thus exerts a radiosensitive effect, we performed a series of rescue experiments. Firstly, we transfected PRDX1 overexpression plasmid into IRAK1-deficient glioma cells, which was verified by Western blotting (Fig. 6a). Colony formation assay showed that PRDX1 overexpression markedly salvaged the survival fraction in IRAK1-deficient cells and reversed the radiosensitizing effect caused by IRAK1 depletion (Fig. 6b, c, Supplementary Fig. S4a, b). Then, we conducted a comet assay to evaluate the extent of DNA damage remaining at indicated time points following IR in sh-NC + Vector, sh-IRAK1 + Vector, and sh-IRAK1 + PRDX1-OE grouped U251 and A172 cells. As observed in Fig. 6d, the levels of DSB represented by comet tail intensity gradually returned to baseline in control (sh-NC + Vector) cells 24 h after IR. Furthermore, IRAK1 knockdown in glioma cells significantly induced the percentage of DNA tail in response to IR treatment at both 30 min and 24 h time points, indicating there exist delays in DNA damage repair in the IRAK1-deficient cells. Notably, PRDX1 overexpression markedly reversed IR-induced higher DNA tail percentage in IRAK1 silencing cells, suggesting that IRAK1 depletion impedes DSB repair by downregulating PRDX1. Consistent with the above data, knockdown of IRAK1 remarkably enhanced the formation of γ-H2AX foci at 24 h after IR treatment, which could be largely reverted by PRDX1 overexpression (Fig. 6e). Together, all these results suggested that the radioresistance of glioma cells is impaired by IRAK1 knockdown, which could be reversed by overexpression of PRDX1.

### PRDX1 silencing increases the radiosensitivity of glioma cells by inducing autophagic cell death

**Fig. 7:**
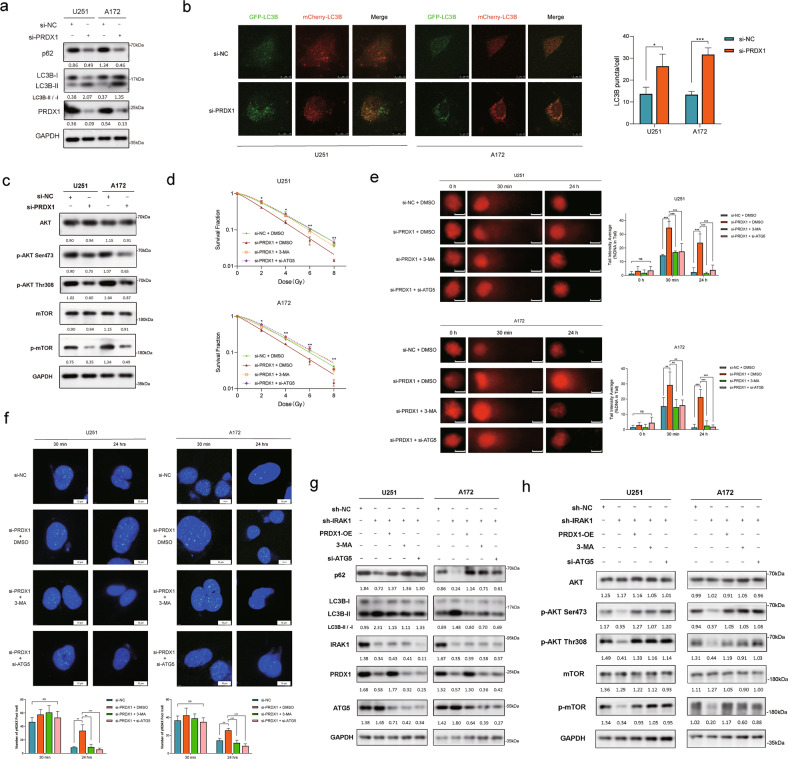
PRDX1 silencing promotes the radiosensitivity of glioma cells via inducing autophagic cell death. **a** The protein levels of p62, LC3B, and PRDX1 in U251 and A172 cells with PRDX1 knockdown were detected by Western blotting assay. **b** U251 and A172 cells with PRDX1 knockdown were transfected with mCherry-GFP-LC3B adenovirus for 48 h to assess autophagic flux by confocal microscopy, and the LC3B puncta was quantified. Scale bar, 10 μm. **c** The protein levels of Akt, p-Akt (Ser473), p-Akt (Thr308), mTOR, and p-mTOR were detected by Western blotting assay. **d** Survival fraction curves of U251 and A172 cells transfected with si-PRDX1 with or without 3-MA/siATG5 treatment (2.5 mM) 24 h before being exposed to 0, 2, 4, 6, 8 Gy of X-rays. **e** Representative images and quantitative analysis of comet tail in the indicated groups of U251 and A172 cells following 8 Gy IR. Scale bar, 10 μm. **f** Cells were transfected with si-PRDX1 or si-NC for 24 h and then treated with 3-MA (2.5 mM) or transfected with si-ATG5 plasmids for another 24 h before being exposed to 8 Gy X-rays. Typical images and the quantification analysis of γ-H2AX foci were presented. Scale bar, 10 μm. IRAK1 silencing U251 and A172 cells were transfected with PRDX1-OE plasmids, si-ATG5 plasmids, or treated with 3-MA (2.5 mM, 24 h). The protein levels of p62, LC3B (**g**), Akt, p-Akt (Ser473), p-Akt (Thr308), mTOR, and p-mTOR (**h**) were detected via Western blotting. Positions of LC3-I and LC3-II are indicated. The LC3-II/LC3-I ratio was calculated based on densitometry analysis of both bands. The gray value ratios of other proteins/GAPDH were shown below each lane. Data were presented as the mean ± SD; *n* = 3 independent experiments. *p < 0.05, **p < 0.01, ***p < 0.001.

PRDX1 has the ability of ROS elimination and scavenge H_2_O_2_ by sacrificing its cysteinyl thiols. Many studies suggested that ROS promotes autophagy execution and affects almost all processes from autophagy induction to autophagy maturation [37]. Thus, we next investigated the effect of PRDX1 on autophagy in glioma cells. As shown in Fig. 7a, U251 and A172 cells with PRDX1 knockdown exhibited higher LC3B-II and lower p62 expression than control cells. In contrast, the level of LC3B-II was downregulated, but the p62 level was upregulated after PRDX1 overexpression (Supplementary Fig. S5a). Consistently, the detection of autophagy flux indicated by the formation of LC3B puncta revealed that the autophagy level was significantly increased in glioma cells with PRDX1 knockdown and decreased in cells with PRDX1 overexpression (Fig. 7b, Supplementary Fig. S5b, c). Given that Akt-mTOR signaling suppressed autophagy [38], we then found that PRDX1 knockdown inhibited the phosphorylation of Akt and mTOR (Fig. 7c).

Having proved that PRDX1 is involved in inhibiting the formation of autophagosomes, we then evaluated whether such regulation is the key mechanism for PRDX1’s impact on the radiosensitivity of glioma cells. 3-Methyladenine (3-MA) is an inhibitor of class III PI3K, VPS34, and disturbs autophagosome formation [39]. Autophagy-related gene 5 (ATG5) is essential for autophagosome formation and the ATG12–ATG5–ATG16 complex acts as an E3 enzyme to catalyze the conversion from LC3B-I to LC3B-II [40]. Therefore, we conducted rescue experiments in si-PRDX1 cells by using the autophagy inhibitor 3-MA or si-ATG5 plasmid simultaneously. The silencing efficiency of ATG5 was verified by Western blotting (Supplementary Fig. S5d). ATG5 silencing inhibited the level of autophagy triggered by PRDX1 knockdown (Supplementary Fig. S5e). The results of clonogenic survival assays revealed that 3-MA treatment or si-ATG5 markedly reversed the downregulated survival fraction resulting from PRDX1 knockdown in U251 and A172 cells (Fig. 7d, Supplementary Fig. S5f). In addition, the percentage of DNA tail in cells irradiated with 8 Gy X-rays at both 30 min and 24 h was observably higher after PRDX1 knockdown than that in the control group, which could be significantly attenuated by 3-MA treatment or si-ATG5 (Fig. 7e). As anticipated, 3-MA treatment and si-ATG5 remarkably reverted the sustained presence of γ-H2AX foci at 24 h after IR treatment induced by PRDX1 knockdown (Fig. 7f). These findings strongly revealed that PRDX1 knockdown enhances glioma cells’ radiosensitivity, at least in part, by inducing autophagic cell death. Interestingly, the increased expression of LC3B-II and the decreased levels of p62 in IRAK1 knockdown cells were pronounced in cells combined with 8 Gy IR (Supplementary Fig. S5g). Overexpression of PRDX1 could weaken IRAK1 depletion-induced autophagy, indicated by decreased LC3B-II and increased p62 expression levels, reaching similar effects with 3-MA treatment or ATG5 silencing (Fig. 7g). The reduced phosphorylation levels of Akt and mTOR in IRAK1 depletion glioma cells were restored by PRDX1 overexpression, ATG5 knockdown or 3-MA treatment (Fig. 7h). Taken together, these results suggested that IRAK1 promotes the radioresistance of glioma cells by PRDX1-mediated suppression of autophagic cell death.

### Suppression of IRAK1 facilitates the radiosensitivity of glioma cells in vivo

**Fig. 8:**
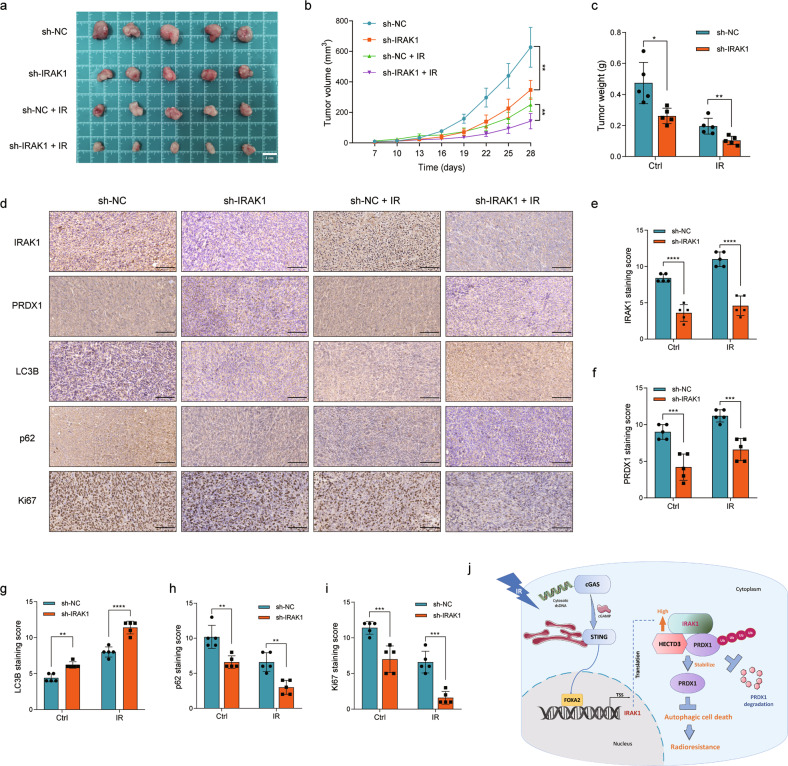
IRAK1 depletion enhances the radiosensitivity of glioma cells in vivo. Typical images of xenograft tumors (**a**), tumor volume (**b**), and tumor weight (**c**) of U251 cells stably transfected with sh-NC or sh-IRAK1 and exposed to IR treatment or not. Representative images (**d**) detected by IHC assay and the corresponding staining score for IRAK1 (**e**), PRDX1 (**f**), LC3B (**g**), p62 (**h**), and Ki67 (**i**) protein expression in the excised tumors from indicated groups (sh-NC/sh-IRAK1/sh-NC + IR/sh-IRAK1 + IR). Scale bar, 100 μm. **j** Proposed working model of the study. IR-stimulated STING activation upregulated the transcription factor FOXA2 expression, and FOXA2 directly binds to the IRAK1 promoter region to initiate its transcription. Increasing IRAK1 expression suppresses autophagic cell death via HECTD3-mediated ubiquitination and degradation of PRDX1, providing a novel mechanism by which IRAK1 overexpression facilitates secondary radioresistance of glioma cells.

To determine whether IRAK1 depletion promotes the radiosensitivity of glioma cells in vivo, we generated U251-derived xenograft nude mice models. The tumor volume and weight of the sh-IRAK1 group were significantly lower than those in the control group, especially following IR treatment, suggesting that the tumors in the IRAK1 depletion group exhibited much more radiosensitivity (Fig. 8a–c). The IHC assays revealed that the protein levels of IRAK1, PRDX1, and Ki67 were decreased after IRAK1 knockdown (Fig. 8d–f, Fig. 8i). Meanwhile, the protein levels of LC3B were significantly increased, and the levels of p62 were decreased in the tumors of sh-IRAK1 group, especially combined with IR treatment (Fig. 8d and Fig. 8g, h). These results indicated better tumor-killing effects of the combination of IRAK1 inhibition and IR treatment in vivo, which is associated with the induction of autophagic cell death.

## Discussion

Elevated expression of IRAK1 has been reported in a variety of tumors, including lung cancer [41], endometrial carcinoma [42], and HCC [10], and has been identified to be correlated with tumor aggressive and poor patient prognosis. However, the role of IRAK1 in glioma has not yet been defined. Previously, our group conducted a comprehensive pan-cancer analysis of the IRAK family and identified IRAK1 as a novel oncogene in low-grade glioma [43]. In this manuscript, we found that IRAK1 expression is elevated in glioma patients from our department, TCGA, and Rembrandt cohorts. Moreover, glioma patients with higher IRAK1 expression exhibited a shorter survival time. Subsequently, we observed that IRAK1 knockdown markedly impedes glioma cell proliferation, migration, and invasion in vitro.

One of the important findings of the study is that the expression level of IRAK1 is upregulated in response to IR treatment. DNA damage-based radiotherapy leads to the accumulation of cytosolic dsDNA in tumor cells, which can be promptly sensed and detected by cGAS. Subsequent binding of dsDNA with cGAS initiates the catalytic synthesis of the second messenger cGAMP, which binds and activates STING [44]. Following transporting from the endoplasmic reticulum to the Golgi complex, the activated STING recruits the TANK-binding kinase 1, triggering the IRF3 and NF-κB for activation [45]. Also, STING activation leads to the induction of type-I IFN. It is widely acknowledged that IRAK1 acts as a classical upstream component in NF-kB pathway and is crucial for inducing IFN-I production. Thus, both STING and IRAK1 are indispensable upstream molecules in the induction of type-I IFN and activation of NF-κB. Considering the intimate relationship of STING signaling and IRAK1, we assumed that STING may mediate IR-induced IRAK1 expression. Herein we confirmed that STING inhibitor H-151 significantly decreased IR-induced IRAK1 transcription. Next, we set out to explore how STING affects IRAK1 and the transcription factor (TF) regulated by STING that binds to the IRAK1 promoter to regulate its transcription. Recently, STING was evidently defined to be enriched in the promoter of the TF FOXA2 [30], which was involved in metabolism, homeostasis, embryo development, and cancer [46]. Supportively, we confirmed that STING inhibition decreased FOXA2 expression. As a member of the forkhead class of DNA-binding proteins, FOXA2 was verified to regulate the transcription of carnitine palmitoyltransferase 2 (CPT2) [47], ciliary neurotrophic factor [48], receptor-interacting protein kinase 3 [49], and multidrug resistance protein 2 [50]. We demonstrated that knockdown of FOXA2 reduced IRAK1 transcription. The ChIP assay and dual luciferase reporter assay confirmed two potential FOXA2-binding sites on the IRAK1 gene promoter predicted by using the JASPAR database. Findings from the current study identified a novel relationship among STING, FOXA2, and IRAK1, providing new insights into the mechanism of the gain of IRAK1 expression upon irradiation. Specifically, the TF FOXA2 is upregulated by IR-stimulated STING activation and directly binds to the IRAK1 promoter region (−1321~−1247 bp, −642~−569 bp) to initiate its transcription.

The anti-tumor response induced by IR relies on innate and adaptive immunity, in which host STING was generally acknowledged to play a pivotal role [51]. However, recent research differed from previous investigations and surprisingly found that IR-elicited STING activation drives extrinsic radiation resistance by enhancing suppressive inflammation due to MDSC infiltration [52]. Besides, the activation of the cGAS-STING-CCL5 pathway in mesenchymal stromal cells mediates IR-induced cancer lung metastasis [53]. Likewise, our clonogenic survival assay showed that STING inhibition markedly downregulated the survival fraction of glioma cells receiving 4 Gy irradiation (Supplementary Fig. S6), supporting the finding of the radiation resistance driven by IR-elicited STING activation. Our findings uncovered that IRAK1 knockdown promotes G2/M phase arrest, apoptosis, especially with IR treatment, and radiosensitization of glioma cells. Based on our results mentioned above, we assumed that IRAK1 induced by IR might be a significant contributing factor to the development of secondary radioresistance in response to STING activation in irradiated tumors, which warrants further intensive studies.

As a core effector of IL-1R/TLR-mediated innate immunity, IRAK1 was reported to counter IR-induced cell death mediated by the PIDDome complex in zebrafish and several human cell models [11]. Herein, our investigation also confirmed that IRAK1 knockdown sensitizes glioma cells to radiation. Therefore, IRAK1 acts as an effector of IR-induced anti-tumor immunity on one hand, and meanwhile as a driver of intrinsic tumor radioresistance. The paradoxical effects of IRAK1 on tumor response would, at first glance, pose therapeutic conundrums. In other words, IRAK1 inhibition would be expected to sensitize tumors to IR but at the expense of thwarting IR-induced anti-tumor immunity. Luckily, kinase-dead IRAK1 was uncovered to retain NF-kB activity, reflecting the strict structure role of IRAK1 protein when engaging TRAF6 to induce NF-kB, rather than relying on its catalytic activity [54]. Thus, the strategy of IR + highly specific IRAK1 inhibitor, whether alone or in combination with STING agonist, would be expected to sensitize tumor cells to IR while also allowing IL-1R/TLR-initiated immune attacks to proceed, which makes our studies focusing on the mechanism of IRAK1 in radioresistant tumors more valuable and promising.

To obtain further insight into the detailed molecular mechanism by which IRAK1 facilitates the delay of DSB response, we screened the interacting proteins using IP coupled to LC-MS/MS proteomics with IRAK1 as the bait. PRDX1 is a typical 2-cysteines peroxiredoxin and acts as an endogenous antioxidant enzyme to scavenge H_2_O_2_ [55]. In the present work, we identified that PRDX1 is a directly interacted protein of IRAK1. Overexpression and the oncogenic function of PRDX1 were demonstrated in many cancers, including breast cancer [56], non-small-cell lung carcinoma [57], pancreatic ductal adenocarcinoma (PDAC) [58], and colorectal cancer [59]. In the studies reported so far, the impact of PRDX1 on autophagy still remains largely elusive and poorly understood. Hajar et al. found that PRDX1 ablation led to reduced autophagic flux in PDAC cells, which is necessary for their survival [58]. Also, the functional role of PRDX1 in NF-κB activation and autophagy activation by inhibiting TRAF6 ubiquitin-ligase activity was uncovered [60]. PRDX1 activates autophagy via the PTEN-AKT signaling to protect against cisplatin-induced spiral ganglion neuron damage [61]. Nevertheless, PRDX1 overexpression was validated to promote the phosphorylation of Akt and inhibit the autophagy flux indicated by diminished LC3B lipidation and LC3B puncta accumulation in HCC [62]. Herein, we demonstrated that PRDX1 effectively leads to enhanced LC3B-II expression and autophagy flux impairment. We propose, to our knowledge, this partial disagreement with some previous studies is mainly attributable to the “double-edged sword” and paradoxical activity of autophagy in cancer. Uncontrolled autophagy activation in cancer evokes excessive “self-digestion” and autophagic programmed cell death [63].

The block of autophagosome-lysosome fusion results in an accumulation of LC3B-II and a reduced autophagic flux, whereas the impairment of autophagosome formation disrupts the conversion from LC3B-I to LC3B-II and decreases autophagic flux [64]. We speculated that PRDX1 inhibits the stage of autophagosome formation according to the upregulated LC3B-II and the alleviation of autophagic flux. It is still not established as to whether autophagy plays a protective role or a destructive role in tumor radioresistance to date. Therefore, in the current work, by manipulating autophagy in glioma using 3-MA inhibiting autophagosome formation or transfecting ATG5 siRNA, we discovered that the inhibition of autophagy attenuated the radiation sensitization effects mediated by the silencing of IRAK1-PRDX1 axis. Also, silencing IRAK1-PRDX1 inhibits the survival of irradiated tumor cells accompanied by increased autophagic levels. Therefore, we concluded that autophagy functions as a cell death mechanism in glioma cells, rather than exerts cytoprotective roles. As the guardian of the genome, the critical tumor suppressor p53 activates autophagy, as evidenced by the induction of autophagy genes in response to p53 activation [65]. Our investigations were performed in both p53 wide-type (A172) and p53 mutant (U251) cell models, indicating the effects of IRAK1-PRDX1 on autophagy and radioresistance were widely applicable regardless of p53 status. Ionizing radiation induces IRAK1 expression in parallel with autophagy activation. We observed the effects of IRAK1 knockdown on pronounced autophagy flux especially combined with IR treatment, validating the autophagy inducer role of IR at the same time. Based on our findings, it is reasonable to conclude that IR-induced IRAK1 overexpression markedly attenuating the autophagic cell death stimulated by IR contributes to the development of secondary radioresistance in glioma.

Our current findings revealed that IRAK1 knockdown facilitates the ubiquitination and degradation of PRDX1. We thus speculated that there might be an intermediate molecular between IRAK1 and PRDX1 that is responsible for the ubiquitination of PRDX1. As predicted by UbiBrowser and IP-MS, we identified an E3-ligase, HECTD3, which mediates the ubiquitination of PRDX1 protein resulting from IRAK1 depletion. HECTD3, a member of the third subfamilies of HECT ligases, was validated to target and ubiquitinate caspase-9, c-MYC, and TRAF3 in cancers, already [66–68]. Interestingly, the analysis of HECTD3 truncated mutants revealed that both full-length, as well as isolated DOC and HECT domains of HECTD3, could interact with PRDX1. In vivo experiments also provide a theoretical basis for more effective tumor control in IRAK1 depletion combined with the IR group. PRDX1 has been previously found to enter into the nucleus and promote the transcription of EMT-related genes [69]. Recent research showed that PRDX1 promotes irradiation induced RAD51 foci formation, protects RAD51 from oxidation, and facilitates homologous recombination (HR) DNA repair [70]. Accordingly, how PRDX1 imports to the nucleus and contributes to repairing DNA damage is still not fully understood and warrants further in-depth elucidation. Figure 8j shows our working model. In summary, the results of our research suggest that IRAK1 could be a potential novel biomarker and radiotherapy desensitizer in glioma. Mechanically, IR induces IRAK1 expression, which was due to direct binding by FOXA2 initiated by IR-elicited STING activation, to suppress autophagic cell death via HECTD3-mediated ubiquitination and degradation of PRDX1, providing a novel mechanism by which IRAK1 overexpression facilitates secondary radioresistance of glioma cells.

## Supplementary information

Supplementary materials

Original Data File

Checklist

## Data Availability

Data and materials are available upon reasonable request if applicable.
