# Supplementary material for: Radiation induces IRAK1 expression to promote radioresistance by suppressing autophagic cell death via decreasing the ubiquitination of PRDX1 in glioma cells
Source: Cell Death Dis. 2023 Apr 8;14(4):259. doi: 10.1038/s41419-023-05732-0 (PMC10082800; doi:10.1038/s41419-023-05732-0)
Supplement: Supplementary file 1 — Supplementary materials [file 41419_2023_5732_MOESM1_ESM.pdf]

Supplementary information

Radiation induces IRAK1 expression to promote radioresistance by suppressing autophagic cell death via decreasing the ubiquitination of PRDX1 in glioma cells

Jing Li et al.,

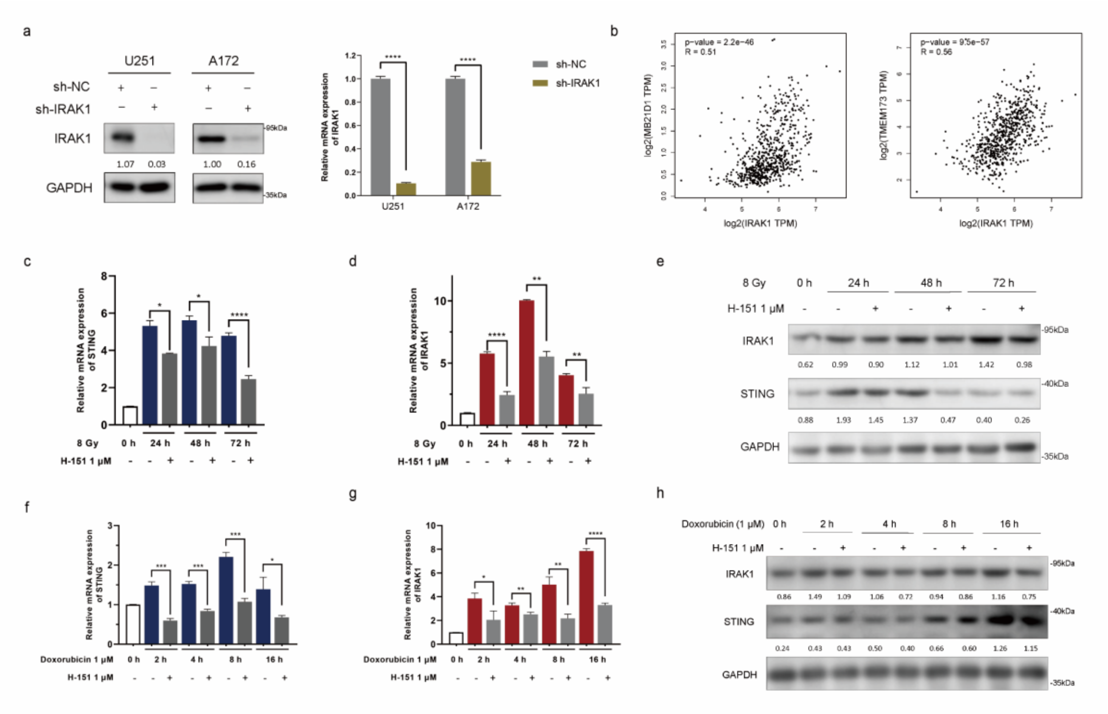

Supplementary Fig. S1

a Western blotting and qRT-PCR analysis of U251 and A172 cells transduced with lentiviral sh-NC or sh-IRAK1. **b** The Spearman correlation coefficients for IRAK1 expression with cGAS (MB21D1) and STING (TMEM173) based on GBM tumor and LGG tumor from TCGA datasets. Analyses were conducted using the GEPIA2 webserver (http://gepia2.cancer-pku.cn/#index). **c, d** Analysis of the mRNA expression of STING (**c**) and IRAK1 (**d**) in A172 cells treated with 8 Gy IR ± H-151 (1 μM) by qRT-PCR. **e** Western blotting analysis to assess the protein expression of IRAK1 and STING in A172 cells at 0, 24, 48, and 72 h after 8 Gy IR with or without H-151 (1 μM) treatment. **f, g**

Analysis of the mRNA expression of STING (**f**) and IRAK1 (**g**) in A172 cells treated with doxorubicin (1 μM) ± H-151 (1 μM) using qRT-PCR. **h** Effects of doxorubicin (1 μM) ± H-151 (1 μM) treatment on IRAK1 and STING protein expression in A172 cells were evaluated by Western blotting. Data were presented as mean ± SD from three independent experiments. **p* < 0.05, ** *p* < 0.01, *** *p* < 0.001, **** *p* < 0.0001.

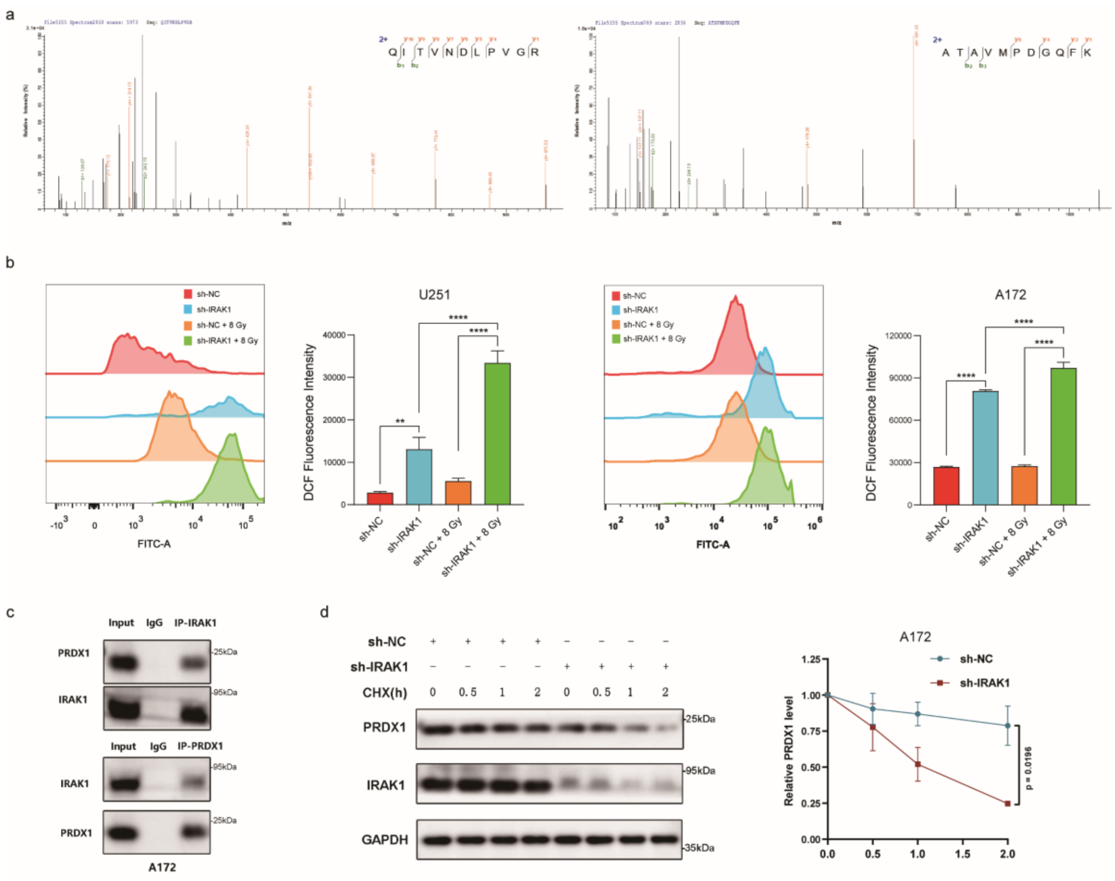

Supplementary Fig. S2

a IRAK1 junction-specific peptides of PRDX1 were identified by IP combined with LC-MS/MS of anti-IRAK1 antibody in U251 cells. **b** Flow-cytometric analysis measured intracellular ROS levels in U251 and A172 cells after IRAK1 stably knockdown, followed by treatment with or without 8 Gy IR. **c** Co-IP detected the interaction of endogenous IRAK1 and PRDX1 in A172 cells. **d** The

effect of CHX treatment (100 μg/mL) and greyscale quantification analysis upon IRAK1 knockdown in A172 cells.

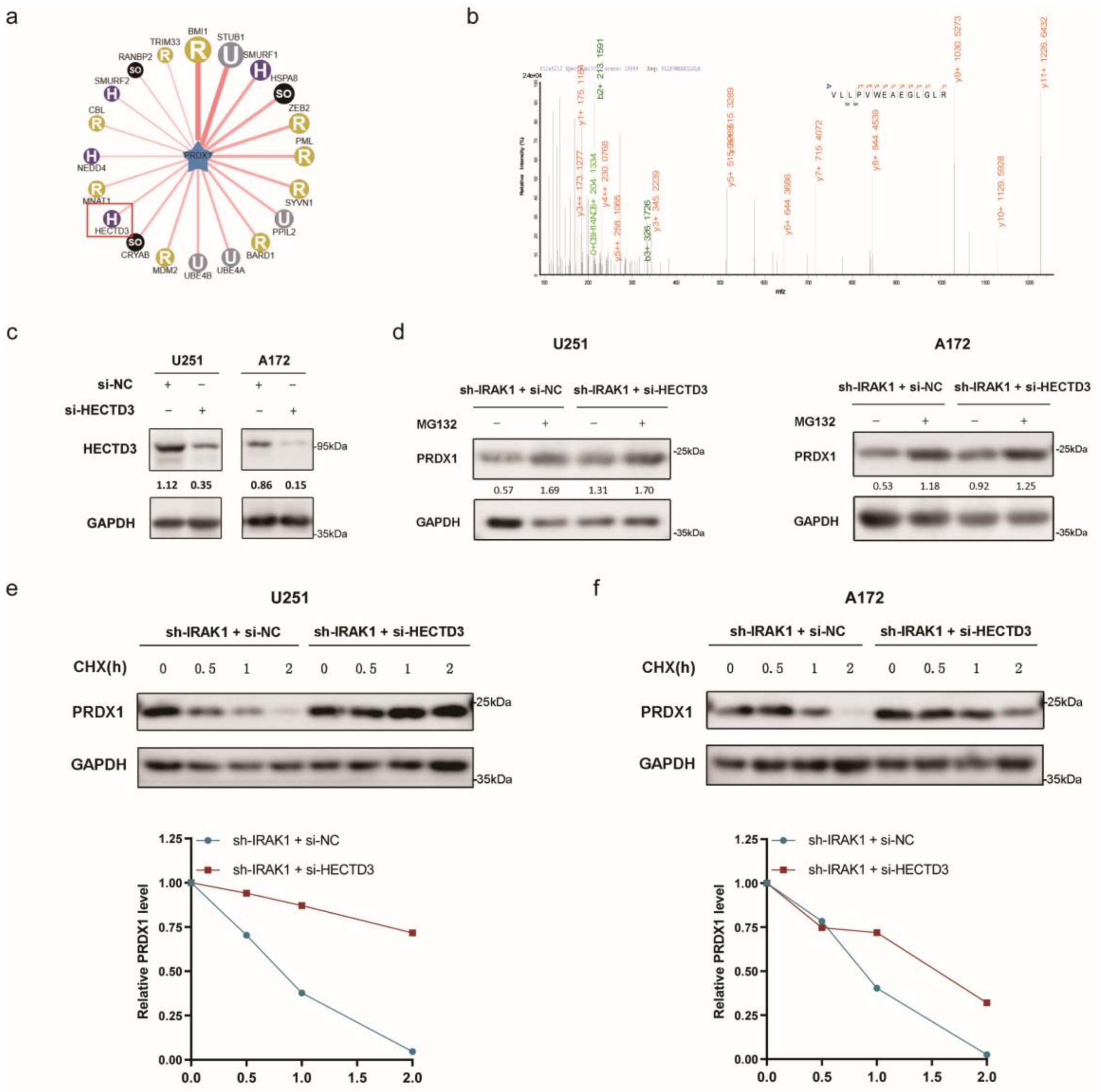

Supplementary Fig. S3

a Prediction of PRDX1 associated E3 ubiquitin ligase by using UbiBrowser webserver. Red rectangle indicated the interested one. **b** Peptides of E3 ligase HECTD3 were identified by IP combined with LC-MS/MS pulled down by anti-IRAK1 antibody in U251 cells. **c** Western blotting assays of U251 and A172 cells transiently transfected with si-NC or si-HECTD3 plasmids. **d** Western blotting detecting the effects of MG132 treatment on the protein levels of PRDX1 in sh-IRAK1 + si-NC and sh-IRAK1 + si-HECTD3 groups. **e, f** Western blotting assays were conducted

to analyze the stability of PRDX1 protein after being treated with CHX (100 μg/mL) for the different duration in sh-IRAK1 + si-NC and sh-IRAK1 + si-HECTD3 groups of glioma cells.

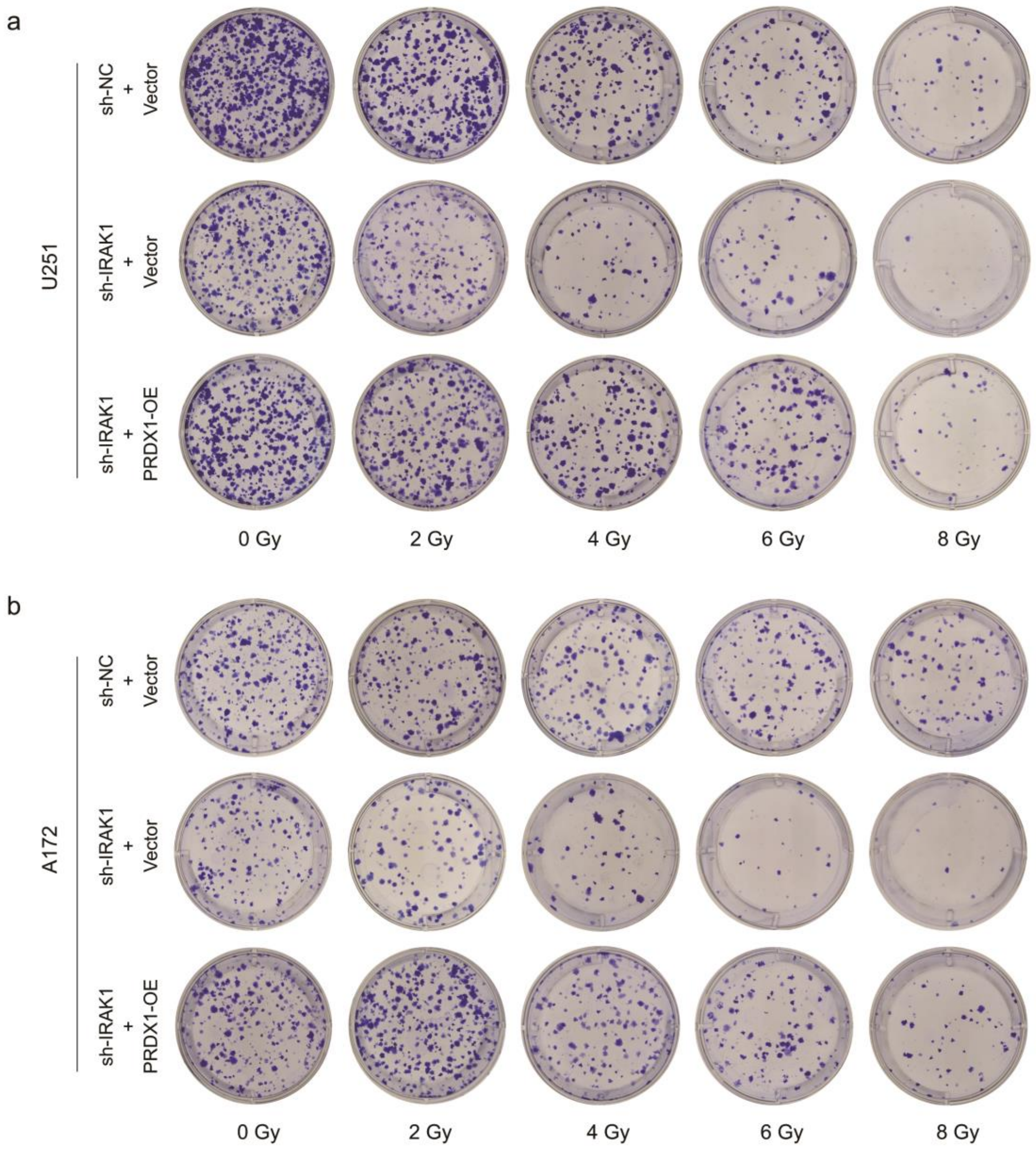

Supplementary Fig. S4

a, b Clone formation assays of sh-NC + Vector, sh-IRAK1 + Vector, and sh-IRAK1 + PRDX1 OE groups based on U251 (**a**) and A172 cells (**b**) following exposure to 0, 2, 4, 6, 8 Gy of X-rays.

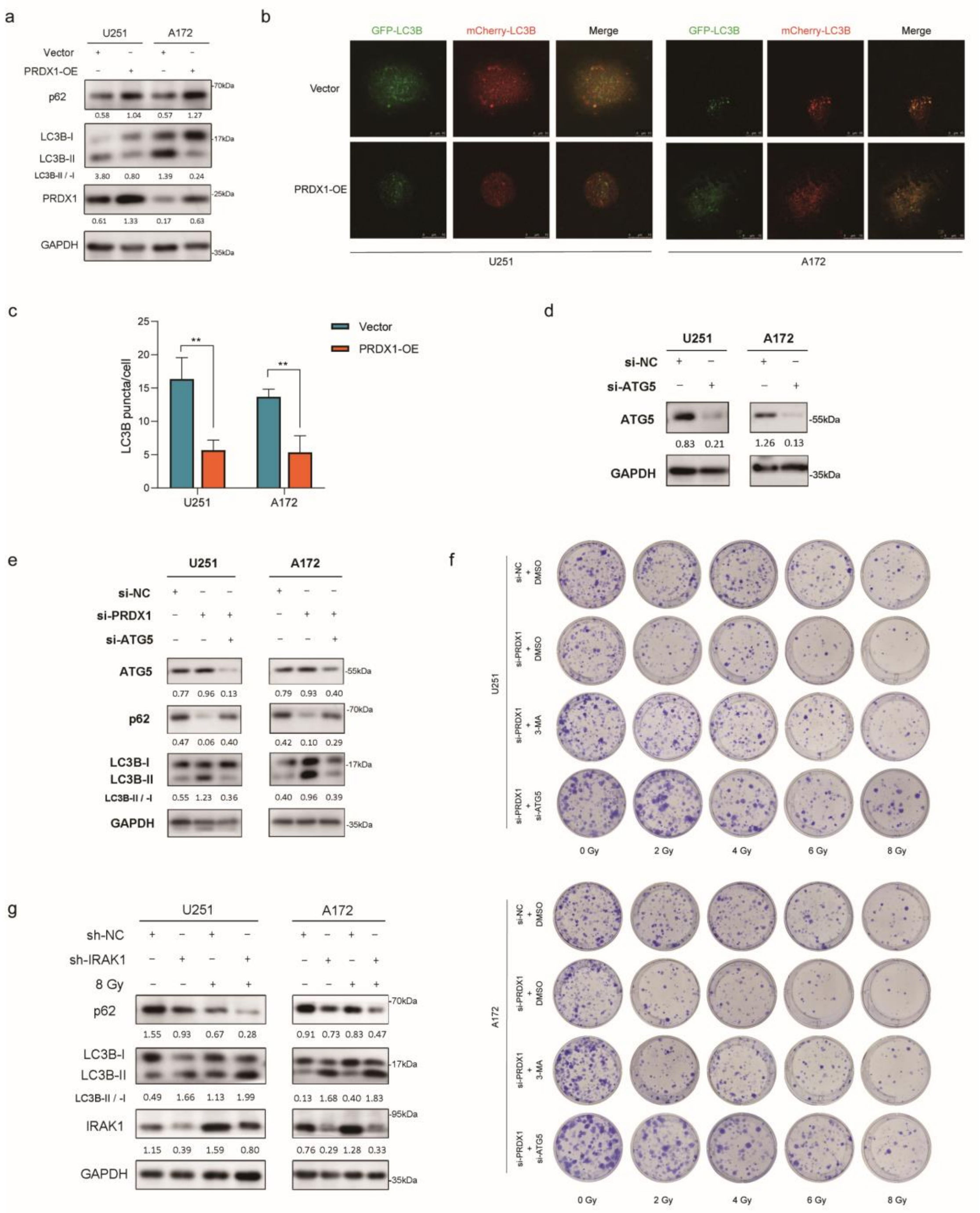

Supplementary Fig. S5

a Western blotting detecting the protein levels of p62, LC3B, and PRDX1 in U251 and A172 cells with PRDX1 overexpression. **b, c** U251 and A172 cells with PRDX1 overexpression were transfected with mCherry-GFP-LC3B adenovirus for 48 h. Autophagic flux was measured by confocal microscopy (**b**), and the LC3B puncta was quantified (**c**). Scale bar, 10 μm. Data were

presented as mean ± SD from three repeats, ** $p < 0.01$. **d** The knockdown efficiency of si-ATG5 in U251 and A172 cells by Western blotting. **e** Western blotting assays detecting the changes of si-ATG5 on the protein levels of p62 and LC3B in PRDX1 silenced cells. **f** Representative images of clone formation assays of U251 and A172 cells transfected with si-PRDX1 with or without 3-MA (2.5 mM, 24 h) or si-ATG5 treatment before exposed to 0, 2, 4, 6, 8 Gy IR. **g** IRAK1 knockdown cells were accepted with 8 Gy IR. Western blotting assays were conducted to detect the protein levels of p62, LC3B, and IRAK1 in different groups. The LC3-II/LC3-I ratio was calculated based on densitometry analysis of both bands. The gray value ratios of other proteins/GAPDH were shown below each lane.

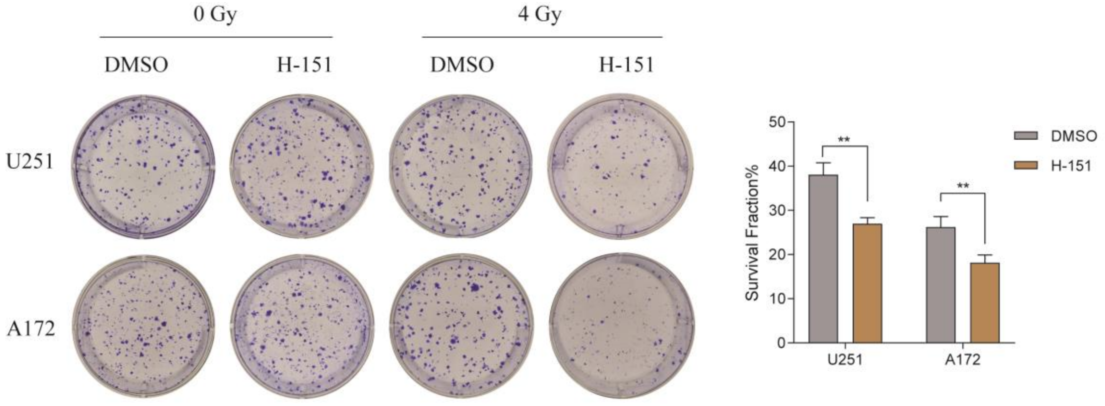

Supplementary Fig. S6

Representative images and the corresponding survival fraction histogram of clone formation assays treated with H-151 (1 μM) or DMSO before being exposed to 4 Gy IR in U251 and A172 cells.

Supplementary Table S1. Radiobiological parameters of IRAK1 knockdown cells and control cells.

	D_0	D_q	N	SF_2
U251				
sh-NC	2.251 ± 0.121	1.311 ± 0.054	1.656 ± 0.171	0.568 ± 0.016

sh-IRAK1	1.360 ± 0.196 **	1.341 ± 0.154	2.925 ± 0.945	0.516 ± 0.017 *
A172				
sh-NC	1.998 ± 0.227	1.043 ± 0.133	1.454 ± 0.363	0.474 ± 0.037
sh-IRAK1	1.586 ± 0.026 *	0.795 ± 0.060 *	1.398 ± 0.096	0.372 ± 0.016 *

Supplementary Table S2. The sequences of lentivirus or plasmids used in this study.

Gene	Target sequences (5'-3')
**shRNA sequences**	
sh-NC	TTCTCCGAACGTGTCACGT
sh-IRAK1	GCCACCGCAGATTATCATCAA
**siRNA sequences**	
si-NC	UUCUCCGAACGUGUCACGUTT
si-PRDX1	ACUCAACUGCCAAGUGAUUTT
si-HECTD3	GCGGGAACTAGGGTTGAAT
si-ATG5	CCAUCAAUCGGAAACUCAUTT
si-FOXA2	GAACGGCATGAACACGTACAT

Supplementary Table S3. List of primers used in this study.

Primers' name		Sequence (5'-3')
**Primers for qRT-PCR**		
IRAK1	Forward	TCAGCTTTGGGGTGGTAGTG
	Reverse	TAGATCTGCATGGCGATGGG
PRDX1	Forward	CTGCCAAGTGATTGGTGCTTCTG
	Reverse	AATGGTGCGCTTCGGGTCTGAT
cGAS	Forward	AAGGATAGCCGCCATGTTTCT
	Reverse	TGGCTTTCAGCAAAAGTTAGG
TMEM173/STING	Forward	AGCATTACAACAACCTGCTACG
	Reverse	GTTGGGGTCAGCCATACTCAG
GAPDH	Forward	GAAGAGAGAGACCCTCACGCTG
	Reverse	ACTGTGAGGAGGGGAGATTCAGT
**Primers for ChIP-qPCR**		
IRAK1-P1	Forward	AAACCTGTCTGAATGTACCTGT
	Reverse	TGCTGCCCATCTTTTCCAAT
IRAK1-P2	Forward	TGGCTCCTCCTGTGTCTCAT
	Reverse	TTGCCTGGTCAGAGAACCAC
IRAK1-P3	Forward	GAGTAAGTCTGCGTTGCTGC
	Reverse	CCTGGGCATCGTCCTTGATT

Supplementary Table S4. List of antibodies used in the study.

Antibody	Company	Catalog no.	Dilution
**Western blot**			
GAPDH antibody, Rabbit Polyclonal	Proteintech	10494-1-AP	1:10000
IRAK1 antibody, Rabbit Polyclonal	Proteintech	10478-2-AP	1:1000
IRAK1 antibody, Rabbit Monoclonal	CST	4504S	1:1000
E-cadherin antibody, Rabbit Polyclonal	Proteintech	20874-1-AP	1:5000
N-cadherin antibody, Rabbit Polyclonal	Proteintech	22018-1-AP	1:2000
Vimentin antibody, Rabbit Polyclonal	Proteintech	10366-1-AP	1:1000
TMEM173/STING antibody, Rabbit Polyclonal	Proteintech	19851-1-AP	1:1000
PRDX1 antibody, Rabbit Polyclonal	Proteintech	15816-1-AP	1:1000
GST Tag antibody, Rabbit Polyclonal	Proteintech	10000-0-AP	1:1000
Ubiquitin antibody, Rabbit Polyclonal	Proteintech	10201-2-AP	1:500
HECTD3 antibody, Rabbit Polyclonal	Proteintech	11487-1-AP	1:2000
LC3B antibody, Rabbit Polyclonal	Proteintech	18725-1-AP	1:1000
Anti-SQSTM1/p62 antibody, Rabbit Monoclonal	Abcam	ab109012	1:2000
AKT antibody, Rabbit Polyclonal	Proteintech	10176-2-AP	1:1000
Phospho-AKT (Ser473) antibody, Rabbit Polyclonal	Proteintech	28731-1-AP	1:1000
Phospho-AKT (Thr308) antibody, Rabbit Polyclonal	Proteintech	29163-1-AP	1:2000
mTOR (7C10) antibody, Rabbit Monoclonal	CST	2983S	1:1000
Phospho-mTOR (Ser2448) (D9C2) XP® antibody, Rabbit Monoclonal	CST	5536S	1:1000
DYKDDDDK (FLAG) Tag antibody, Rabbit Polyclonal	Proteintech	20543-1-AP	1:2000
HRP-labeled Goat Anti-Rabbit IgG (H+L) antibody	Beyotime	A0208	1:2000
HRP-labeled Goat Anti-Mouse IgG (H+L) antibody	Beyotime	A0216	1:1000
Anti-FOXA2 antibody-ChIP Grade, Rabbit Monoclonal	Abcam	ab108396	1:500
ATG5 antibody, Rabbit Polyclonal	Proteintech	10181-2-AP	1:2000
**Immunofluorescence**			
IRAK1 antibody, Mouse Monoclonal	Proteintech	66653-1-Ig	1:100
PRDX1 antibody, Rabbit Polyclonal	Proteintech	15816-1-AP	1:150
IRAK1 antibody, Rabbit Polyclonal	Proteintech	10478-2-AP	1:200
cGAS antibody, Rabbit Polyclonal	Proteintech	26416-1-AP	1:100

TMEM173/STING antibody, Rabbit Polyclonal	Proteintech	19851-1-AP	1:200
Phospho-Histone H2A.X (Ser139) (20E3) antibody, Rabbit mAb	CST	9718S	1:400
Goat Anti-Rabbit IgG, Cy3 Conjugated secondary antibody	zhuangzhibio	EK022	1:100
Goat Anti-Mouse IgG, Cy3 Conjugated secondary antibody	zhuangzhibio	EK012	1:100
Goat Anti-Rabbit IgG, FITC Conjugated secondary antibody	zhuangzhibio	EK023	1:100
**Co-Immunoprecipitation**			
Normal Rabbit IgG	CST	2729S	1:100
IRAK1 antibody, Rabbit Monoclonal	CST	4504S	5 μg
PRDX1 antibody, Rabbit Polyclonal	Proteintech	15816-1-AP	5 μg
HECTD3 antibody, Rabbit Polyclonal	Proteintech	11487-1-AP	5 μg
MYC Tag antibody, Rabbit Polyclonal	Proteintech	16286-1-AP	3 μg
Anti-FLAG® M2 antibody produced in mouse	Sigma	F1804	3 μg
**Chromatin Immunoprecipitation (ChIP)**			
Anti-FOXA2 antibody-ChIP Grade, Rabbit Monoclonal	Abcam	ab108396	1.5 μg
Normal Rabbit IgG	CST	2729S	1.5 μg
**Immunohistochemistry**			
IRAK1 antibody, Rabbit Polyclonal	Proteintech	10478-2-AP	1:600
PRDX1 antibody, Rabbit Polyclonal	Proteintech	15816-1-AP	1:200
LC3B antibody, Rabbit Polyclonal	Proteintech	18725-1-AP	1:400
SQSTM1/p62 antibody, Rabbit Polyclonal	Proteintech	18420-1-AP	1:100
Ki67 antibody, Rabbit Polyclonal	Proteintech	27309-1-AP	1:10000
