# Supplementary material for: Radiation induces IRAK1 expression to promote radioresistance by suppressing autophagic cell death via decreasing the ubiquitination of PRDX1 in glioma cells
Source: Cell Death Dis. 2023 Apr 8;14(4):259. doi: 10.1038/s41419-023-05732-0 (PMC10082800; doi:10.1038/s41419-023-05732-0)

2a

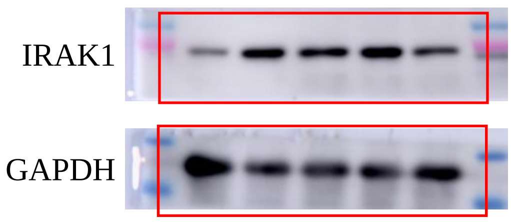

2f

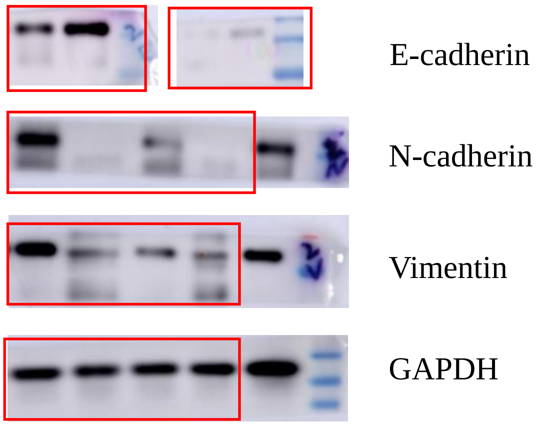

3a

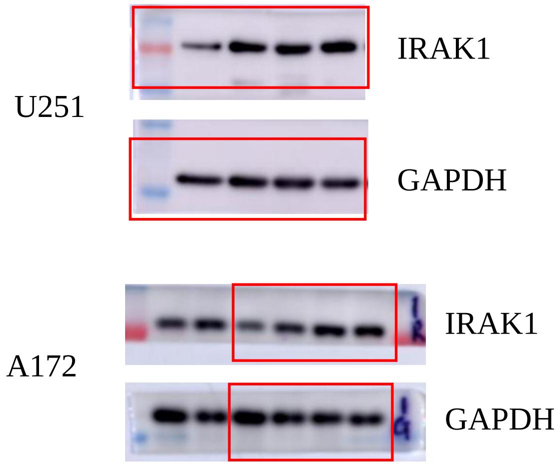

3g

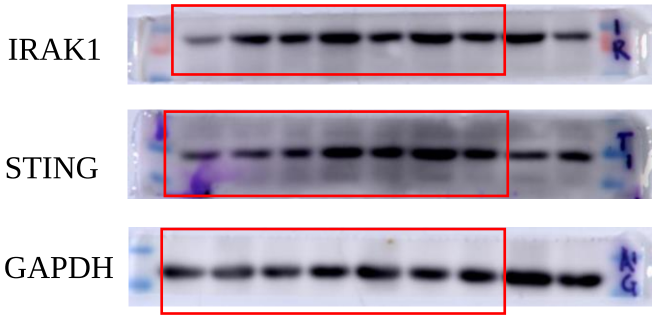

3j

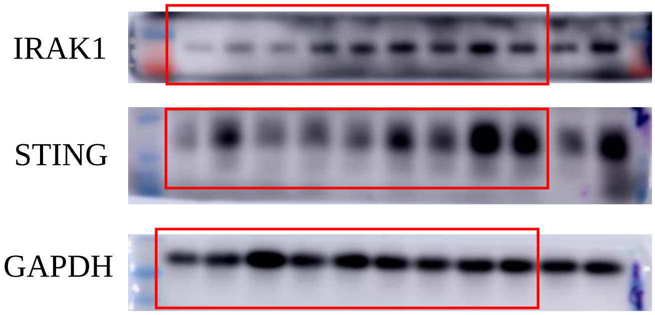

3l

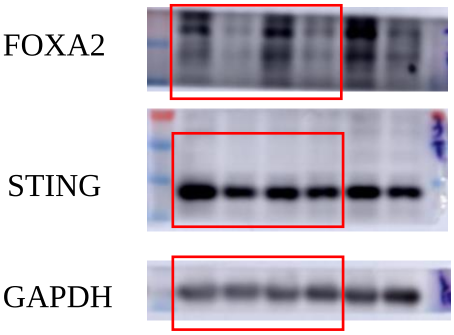

3n

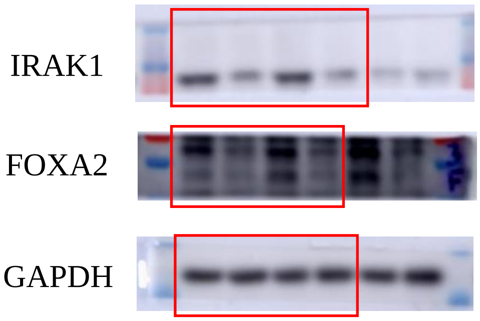

S1a

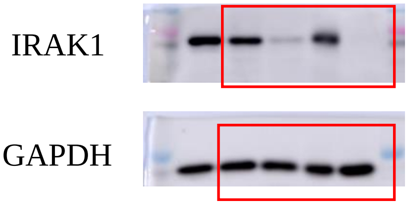

S1e

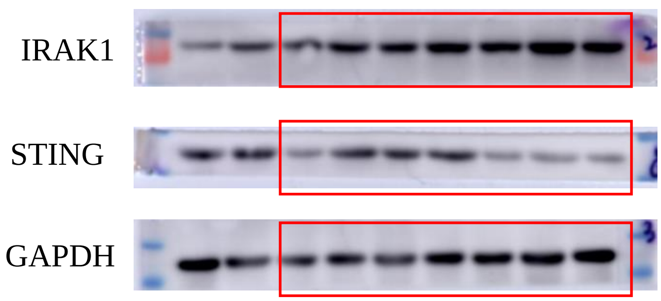

S1h

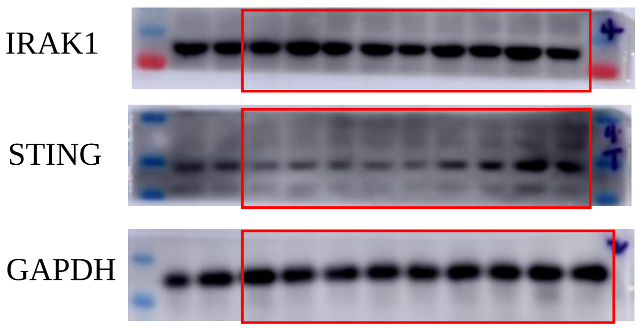

5b

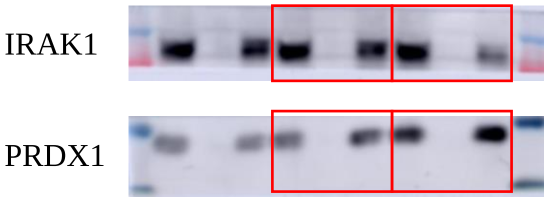

5c

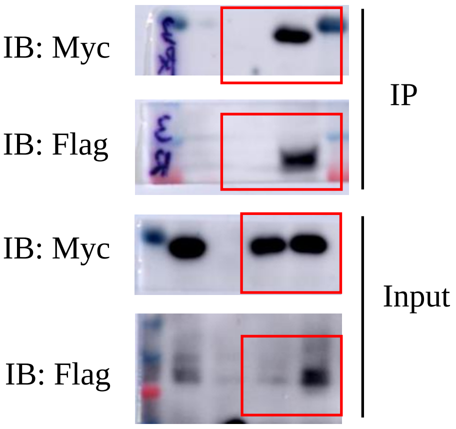

S2c

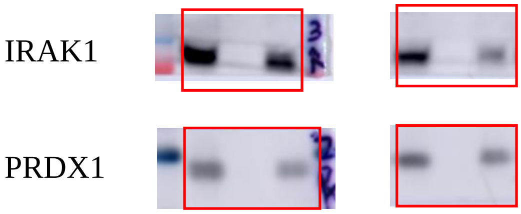

5f

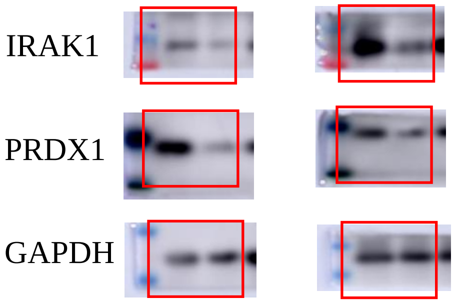

5e

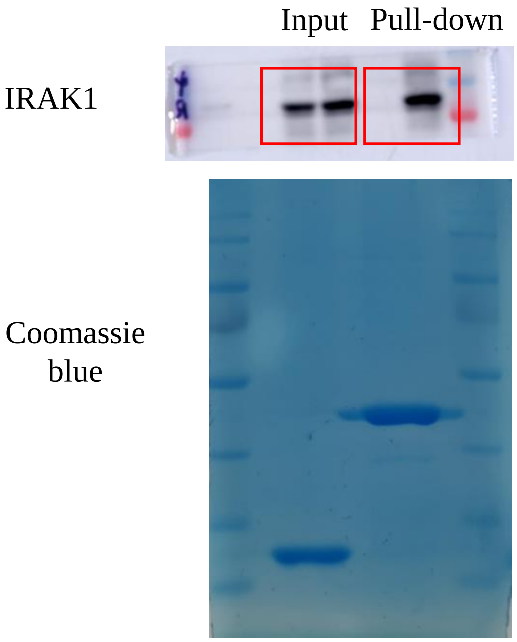

5g

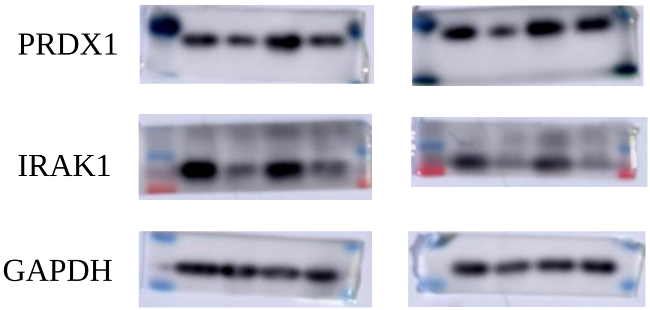

5h

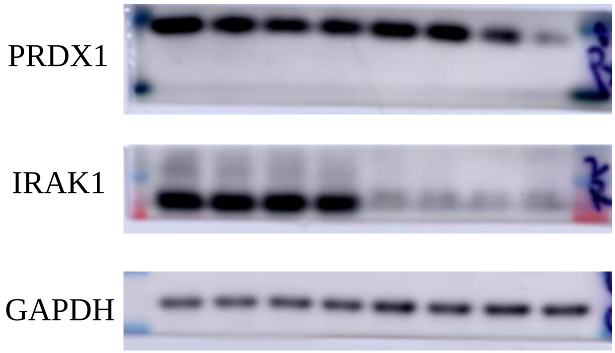

5i

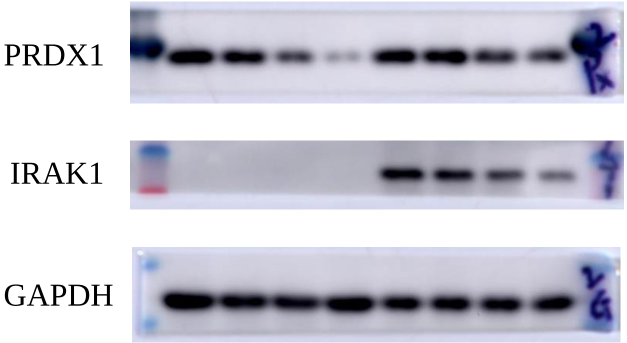

S2d

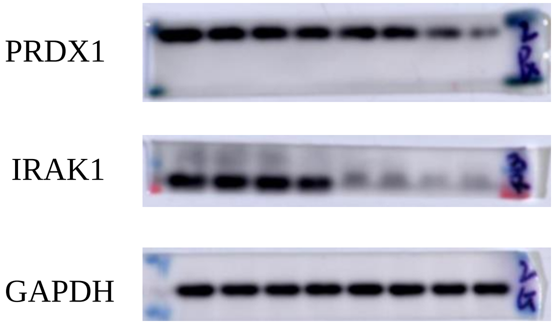

5k

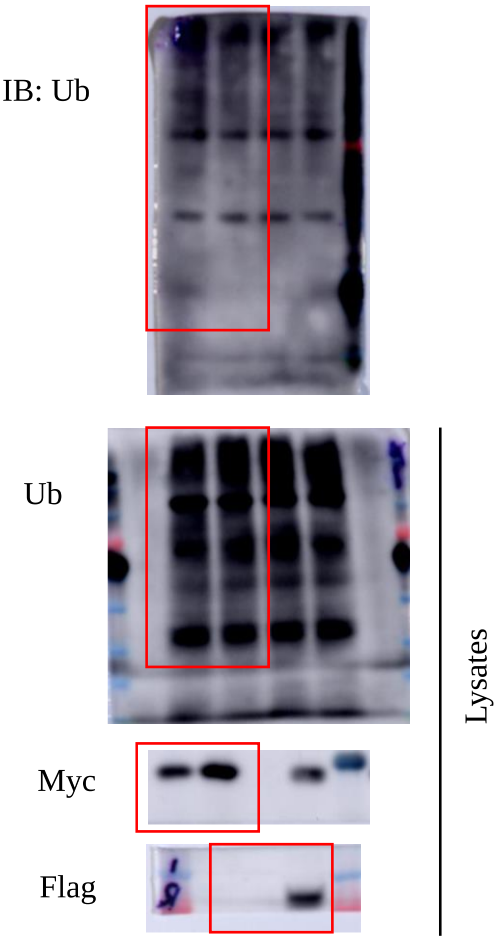

5j

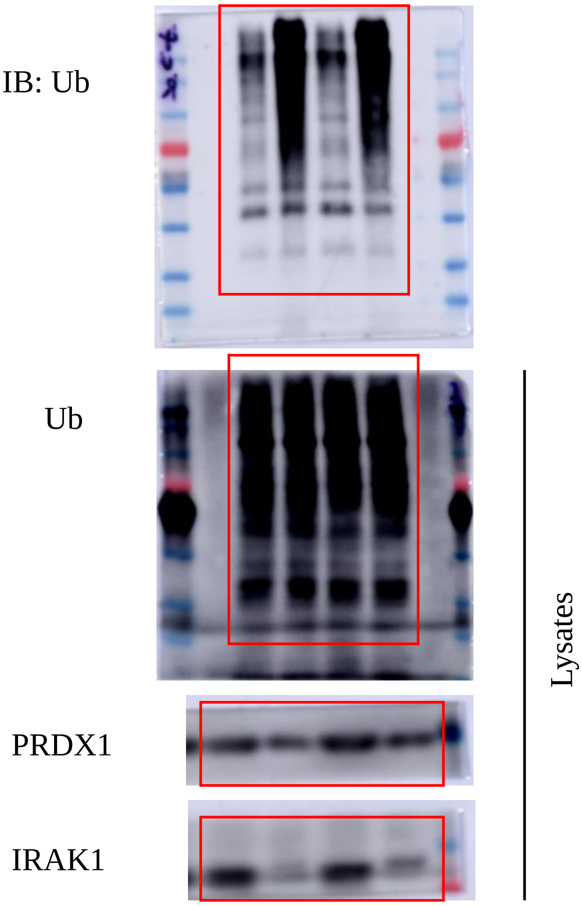

5l

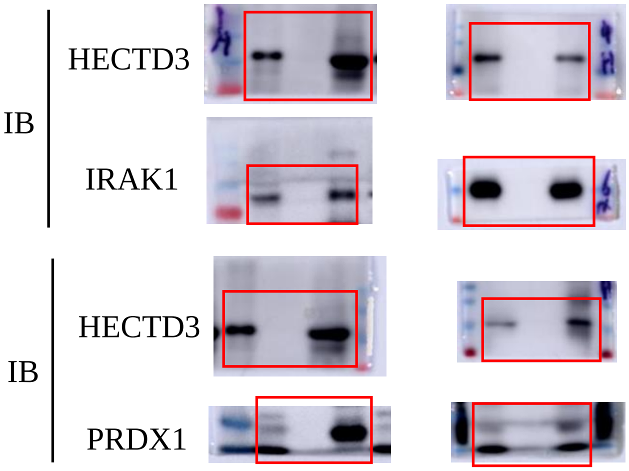

5m

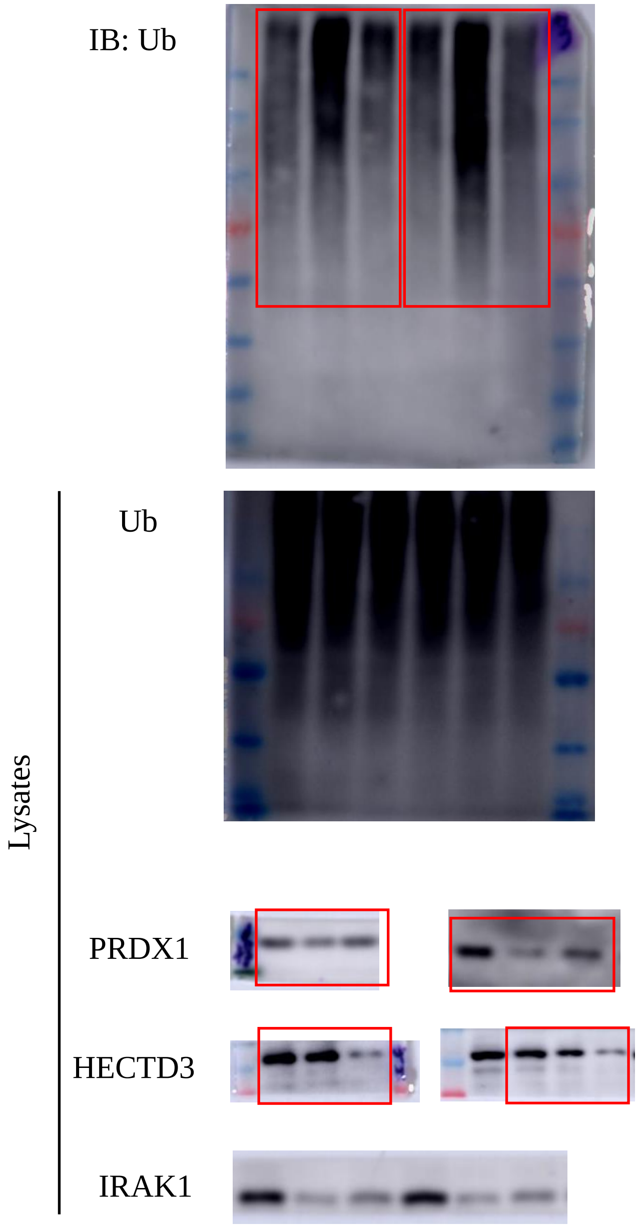

5o

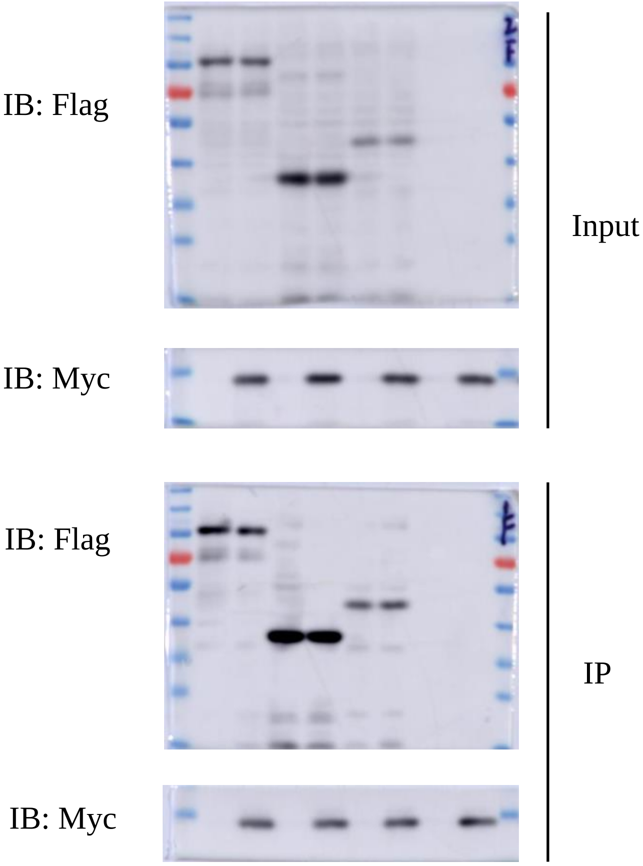

S3c

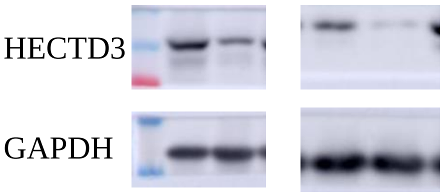

S3d

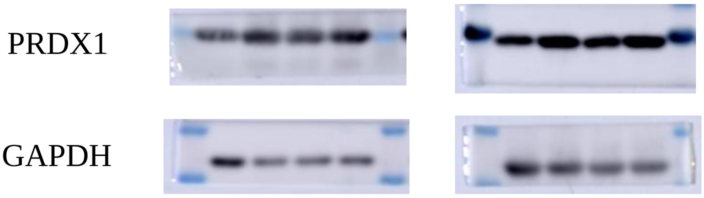

S3e

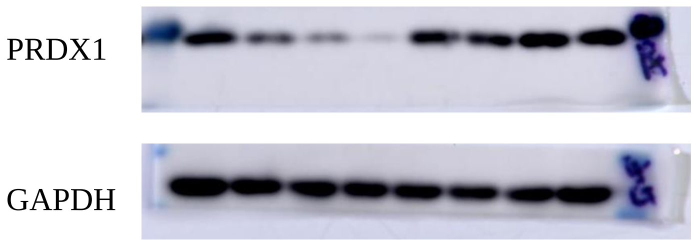

S3f

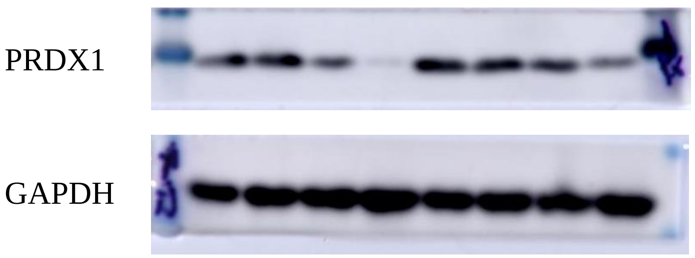

6a

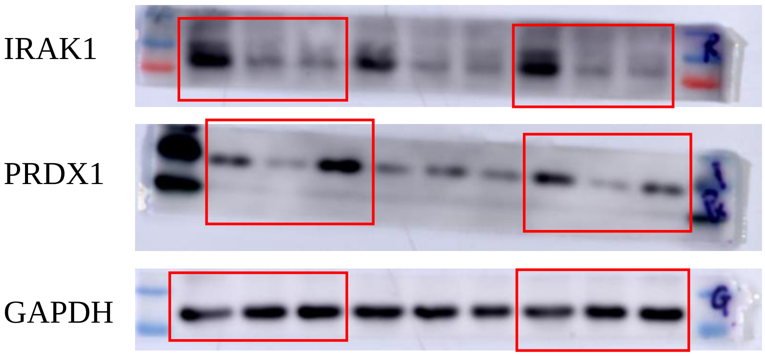

S5d

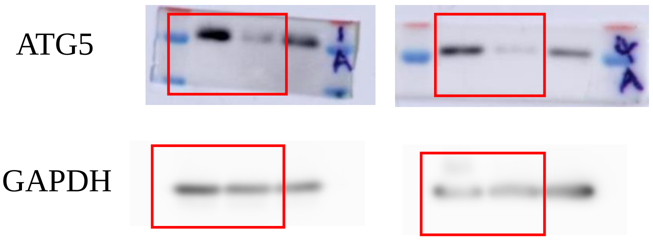

7a

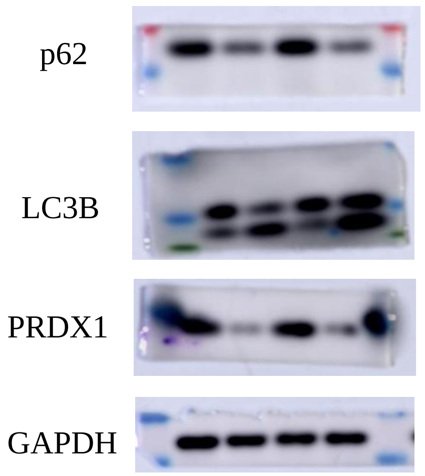

S5a

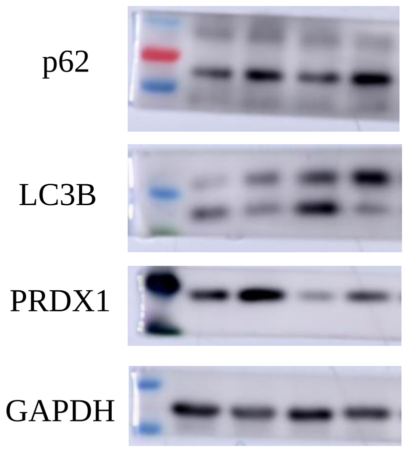

S5e

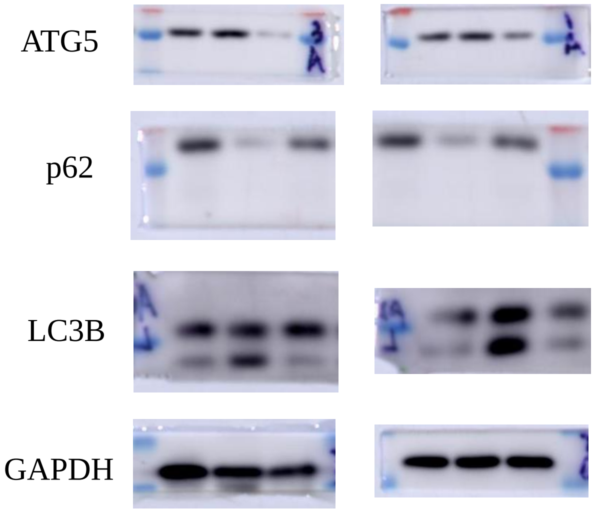

7c

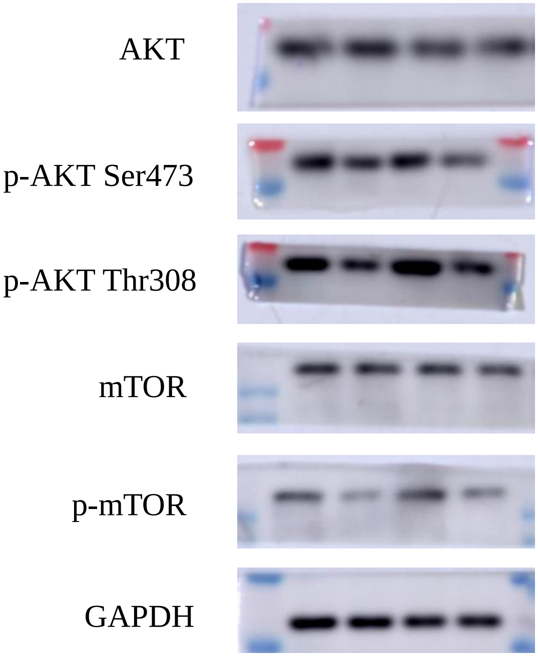
AKT
p-AKT Ser473
p-AKT Thr308
mTOR
p-mTOR
GAPDH

S5g

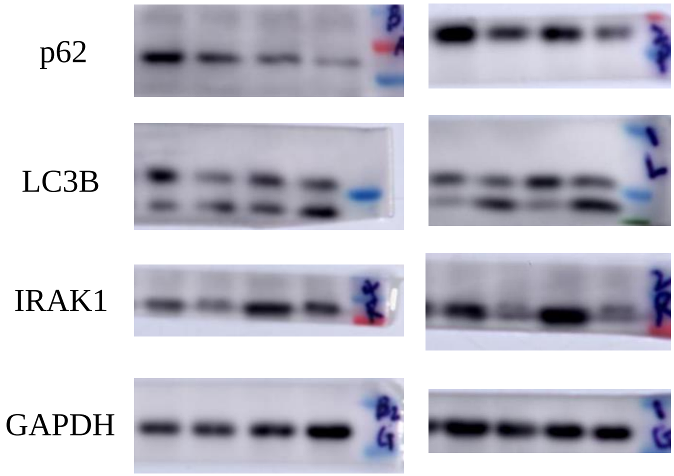
p62
LC3B
IRAK1
GAPDH

7g

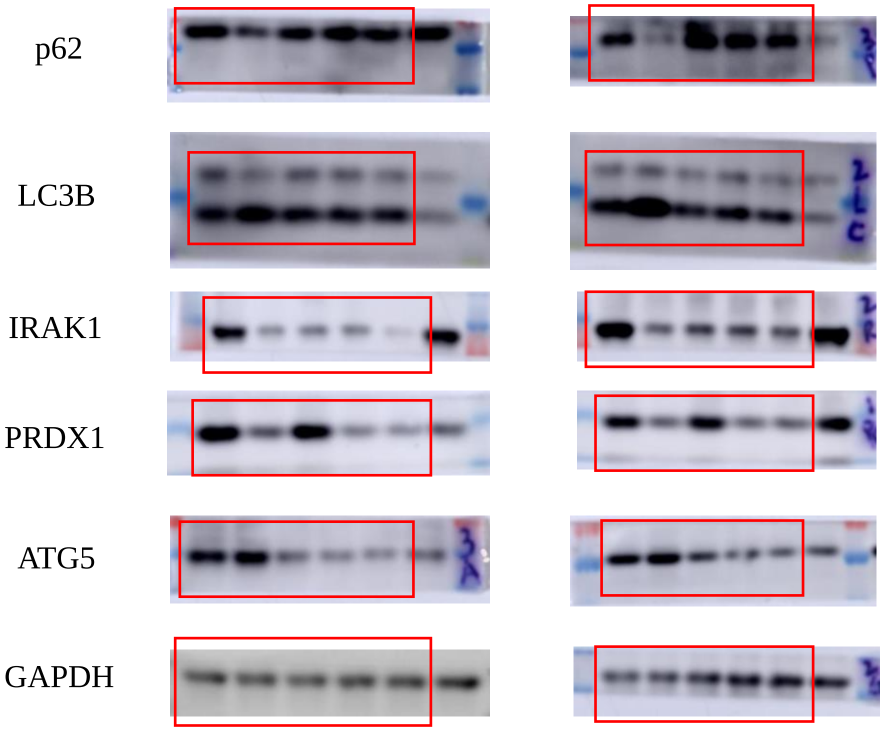
p62
LC3B
IRAK1
PRDX1
ATG5
GAPDH

7h

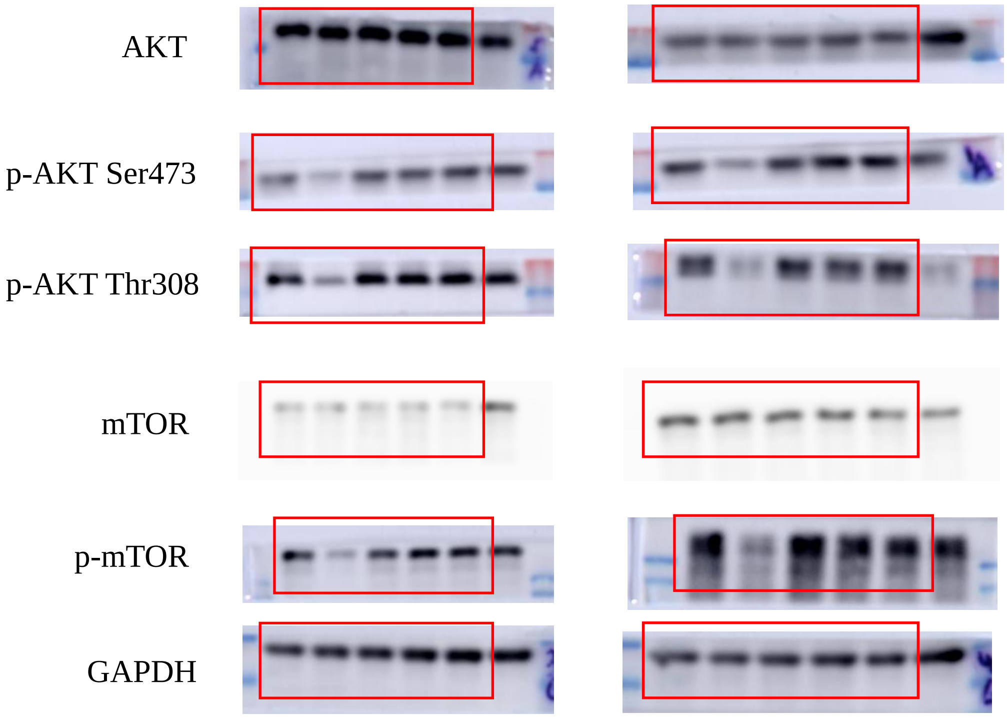

Supplement: Supplementary file 2 — Original Data File [file 41419_2023_5732_MOESM2_ESM.pdf]
